# Long-Range Supramolecular Assembly of a Pyrene-Derivatized Polythiophene/MWCNT Hybrid for Resilient Flexible Electrochromic Displays

**DOI:** 10.1021/acsaenm.4c00534

**Published:** 2024-10-21

**Authors:** Rúben R. Ferreira, Dario Mosca, Tiago Moreira, Vivek Chandrakant Wakchaure, Gianvito Romano, Antoine Stopin, Carlos Pinheiro, Alexander M. T. Luci, Luís M. A. Perdigão, Giovanni Costantini, Heinz Amenitsch, Cesar A. T. Laia, A. Jorge Parola, Laura Maggini, Davide Bonifazi

**Affiliations:** † Institute of Organic Chemistry, University of Vienna, Währinger Straße 38, 1090 Vienna, Austria; ‡ Department of Chemistry and Namur Research (NARC), University of Namur (UNamur), Rue de Bruxelles 61, 5000 Namur, Belgium; § Department of Chemistry, Faculty of Science and Technology, Universidade NOVA de Lisboa, Campus de Caparica, 2829-516 Caparica, Portugal; ∥ School of Chemistry, Cardiff University, Park Place Main Building, CF10 3AT Cardiff, U.K.; ⊥ Ynvisible GmbH, Engesserstr. 4A, 79108 Freiburg, Germany; # Department of Chemistry, University of Warwick, Gibbet Hill Road, CV4 7AL Coventry, U.K.; ¶ School of Chemistry, University of Birmingham, B15 2TT Birmingham, U.K.; ∇ Institute for Inorganic Chemistry, University of Technology, Stremayergasse 9/V, 8010 Graz, Austria

**Keywords:** polythiophene, pyrene, carbon nanotubes, supramolecular nanostructuring, flexible electrochromic displays

## Abstract

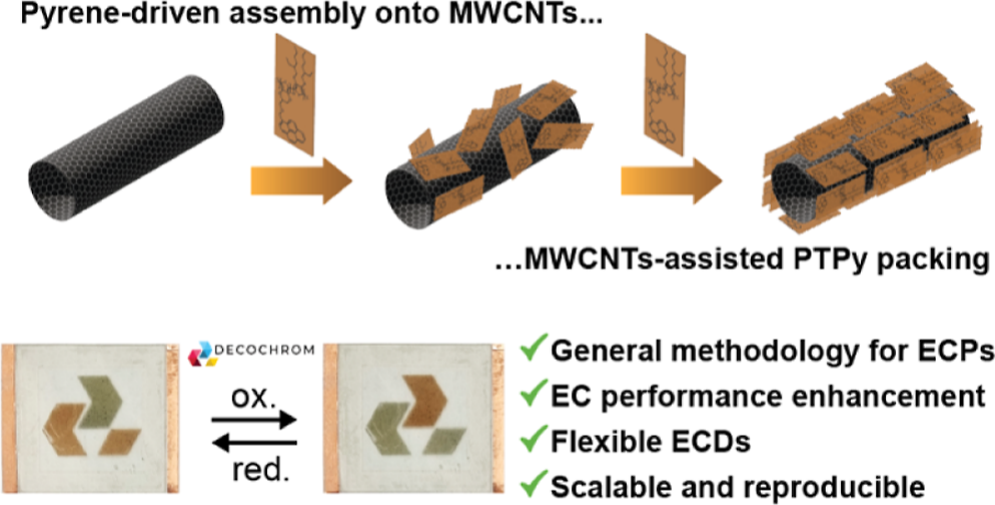

Organic electrochromic polymers hold great potential for integration into low-power flexible electrochromic displays (F-ECDs) due to their wide range of colors and simple processing. However, challenges such as inefficient charge transfer and degradation upon device integration hinder their practical applications. Herein, we report an innovative, general approach that utilizes template-induced supramolecular nanostructuring to engineer established electrochromic polymers, enhancing their performance and durability. We modified a well-known, albeit underperforming in F-ECDs, poly-thiophene polymer (ECP Orange; PT) by incorporating a pyrene appendage, resulting in a copolymer (**PTPy**) capable of undergoing large-scale assembly in the presence of multi-walled carbon nanotubes (MWCNTs), driven by the establishment of π–π interactions between the pyrene and the MWCNTs (**PTPy/MWCNTs**). F-ECDs based on these hybrids, produced by spray coating, exhibit improved color switching speeds (*t*_90_^OX^ = 3.6 s, *t*_90_^RED^ = 0.3 s) compared to those of the **PT** polymer (*t*_90_^OX^ = 53.2 s, *t*_90_^RED^ = 2.5 s). Additionally, **PTPy/MWCNTs** F-ECDs demonstrate longer cyclability (half-life based on Δ*E*, Δ*E*_50%_ = 17.6k cycles) compared to **PT** (Δ*E*_50%_ = 278 cycles), also when blended with MWCNTs (Δ*E*_50%_ = 282 cycles). This work highlights the pivotal role of engineered supramolecular nanostructuring in boosting the performance of organic electrochromic materials, making them suitable for F-ECD scalable commercial applications.

## Introduction

Electrochromic materials (ECMs) can undergo an electrical-bias-induced charge-transfer process, leading to a reversible change in optical absorption. Electrochromic displays (ECDs) that utilize this property have demonstrated significant potential for integration into flexible, deformable, and wearable electronics.^1^ They serve as nonlight-emitting, lightweight, and low-power interactive interfaces applicable to smart textiles, consumer goods, and appliances within the Internet of Things (IoT) vision.^1^ However, incorporating flexibility into ECDs (i.e., F-ECDs) while maintaining effective charge mobility, fast switching, and long cyclability is a challenging task.^2^ Successful integration is closely linked to the ECM’s ability to adapt to any mechanical strain experienced by the flexible substrate. Researchers are actively addressing this challenge by proposing novel engineering paradigms for ECMs, going beyond the blunt boost in their performance in artificial contexts. Devising ECMs that can make F-ECD accessible as a low-energy-consuming sustainable technology^2,3^ still needs to overcome the issues of improving charge and discharge processes and stability in operating conditions.

In this context, Wang and co-workers demonstrated the possibility to enhance the EC performances of 2D transition metal oxides (i.e., TiO_2_) by coassembling them with 2D MXenes (e.g., Ti_3_C_2_T_*x*_) into 2D TiO_2_/Ti_3_C_2_T_*x*_ heterostructures.^3^ These are characterized by flexibility, mechanical stability, and low switching time (0.15–0.76 s), benefiting from the well-balanced porosity and connectivity between the assembled structures. The development of similar assembly strategies for organic ECMs, and especially for the kaleidoscopic palette of conjugated electrochromic polymers (ECPs),^4−8^ would be decisive to finally implement their exploitation in commercial F-ECD devices. Indeed, despite advantages such as colorfulness, quick switching times (<3 s), appreciable light transmittance change (up to 80 Δ%*T*), mechanical flexibility, bistability (up to hours), solution (e.g., spray-coating), and high-throughput (e.g., roll-to-roll) processability,^9,10^ ECPs still suffer poor chemical/cycling stability, adhesion issues, and mostly fail in solid-state devices due to ineffectual charge mobility.^11^ This limitation becomes particularly relevant when thicker films are produced to achieve higher color contrast.^1,12^

In an effort to structure ECPs with enhanced electrochromic performances, Wang and co-workers reported the preparation of hierarchical electrochromic thin films composed of polyaniline particles.^13^ By preorganizing the ECP into particles, mechanical flexibility, higher coloration efficiency (η = 80.9 cm^2^ C^–1^ at 630 nm), and noticeable multicolor performances could be obtained. Laia and co-workers reported films of poly(3-hexylthiophene-2,5-diyl) (P3HT) nanoparticles, presenting improved switching (*t*_90_ of 4 s with 100 nm P3HT-NPs) and cyclability (1k cycles using ±1.5 V, 20 Δ%*T*) when compared to bulk ECPs.^8^ Schott and co-workers described a side chain-modified PEDOT that can be electropolymerized as a porous film in a roll-to-roll slot-die coating polymerization process.^6^ The increased surface area and structuring resulted in an accelerated anion insertion process, providing high coloration efficiency (η = 530 cm^2^ C^–1^), long cycling stability (10k cycles), and fast switching times (<10 s). Recently, Lu and co-workers developed a fluorinated polythiophene with superior electrochromic performance (η = 752 cm^2^ C^–1^), thanks to the formation of structuring intermolecular hydrogen bonds established during the electropolymerization process.^14^

In these pioneering examples, ECP processing requires nanostructuring steps (e.g., particle formation, wetting/drying steps, etc.) during film production, which might translate into scale-up challenges. To the best of our knowledge, a straightforward, general nanostructuring approach that can be readily implemented in industrial process lines to achieve both enhanced performance and enhanced compatibility with F-ECDs is yet to be developed.

**Figure 1:**
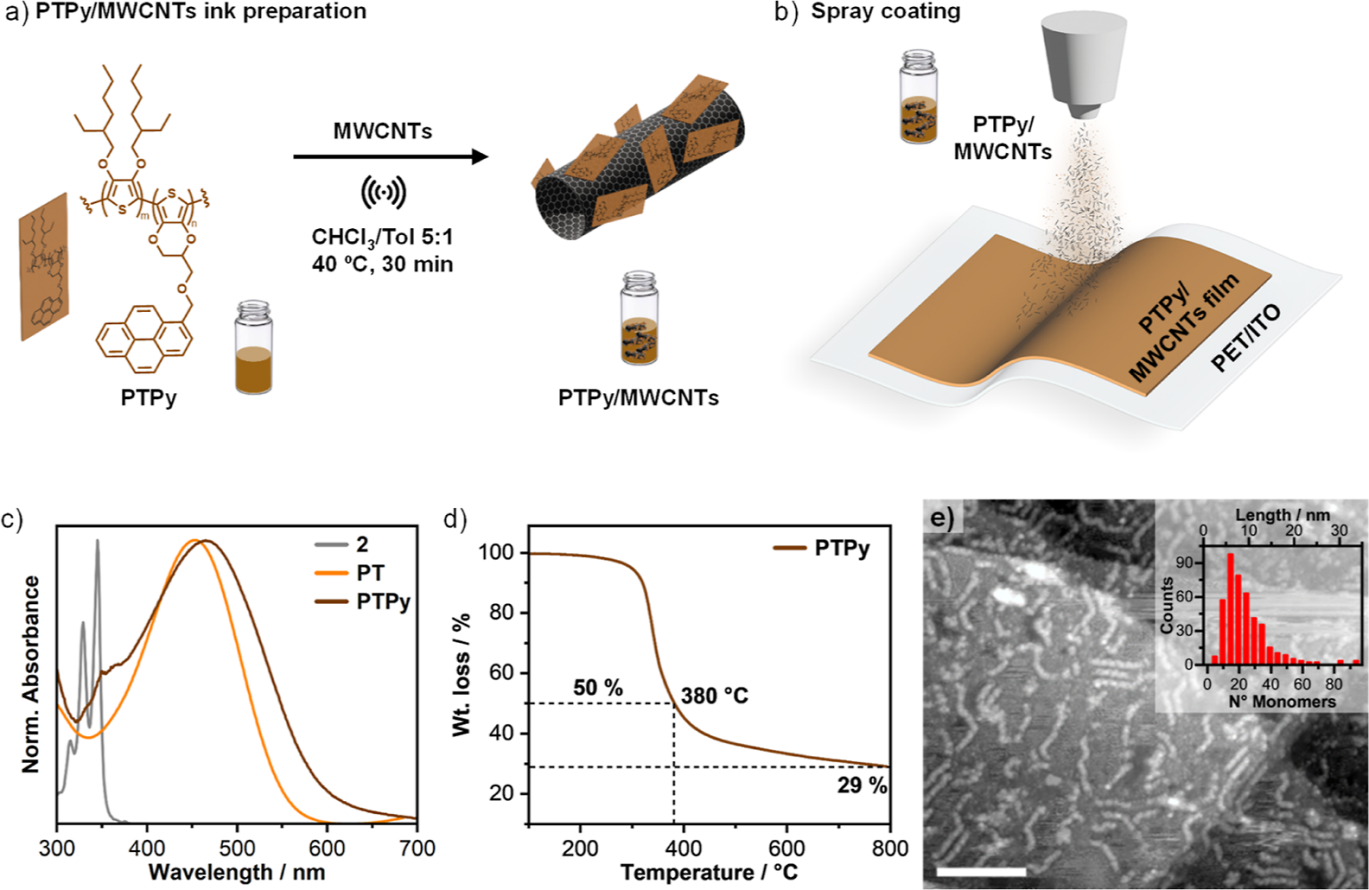
Synthesis and characterization of **PTPy/MWCNT** blends. (a) Chemical structure of **PTPy** and the methodology used for preparing **PTPy/MWCNT** dispersions. (b) Graphical representation illustrating the spray-coating process for **PTPy/MWCNTs**. (c) Normalized UV–vis absorption spectra of **2**, **PT**, and **PTPy** in CHCl_3_. (d) TGA profile of **PTPy** recorded under an inert atmosphere (N_2_, 60 mL min^–1^), with a ramp of 10 °C min^–1^ from 100 to 800 °C. (e) Scanning tunneling microscopy (STM) image and length distribution (inset) of the copolymer **PTPy** obtained by STM analysis performed onto a Au(111)/mica surface; scale bar 20 nm.

In this paper, we present a generalized, template-based long-range supramolecular assembly method for nanostructured ECP films that demonstrates reduced switching times and enhanced cyclability in F-ECDs compared to bulk ECPs. This approach is also compatible with scalable production methods. Capitalizing on the strong affinity between the ECP and a seed template, our approach aims at: (i) reproducibly structuring of the ECP at the nanoscale, (ii) enhancing the charge and discharge processes, and (iii) endowing the ECP with mechanical resilience upon deformation stresses for exploitation in F-ECDs. Building on these premises, we selected multi-walled carbon nanotubes (MWCNTs) as a template due to their extended graphitic structure enabling π–π interactions,^15^ excellent conductivity,^16^ and electron mobility.^17−19^ MWCNTs have been employed to prepare ECP nanocomposites; for instance, Kumar and co-workers fabricated solid-state ECDs with P3HT and ethyl viologen layers predoped with MWCNTs, either exclusively or in combination with MoS_2_.^20^ These nonflexible ECDs exhibited fast color switching times −( 1.0 and 0.5 s for MWCNTs and 0.5 and 0.8 s for MWCNTs/MoS_2_, for the coloration and bleaching processes, respectively−and highcoloration efficiencies (η = 401 and 642 cm^2^ C^–1^) at voltages as low as 1.4 V, stable up to 100 cycles. This approach was also applied to polyaniline^21^ and metallopolymers;^22,23^ however, the intrinsic irreproducibility of these nonengineered nanocomposites, which are based on stochastic polymer adsorption onto the MWCNTs, along with their nonscalable production processes (e.g., spin-coating and drop-casting), has limited their further development and integration into F-ECDs. With the aim of fully leveraging the synergistic potential of the ECP/MWCNT hybrid and achieving precise and consistent control over its structuring, we derivatized a known yet underperforming ECP (i.e., ECP Orange; **PT**) with a pyrene (**Py**) moiety. This **Py**-modified ECP (**PTPy**) was specifically designed to facilitate the anchoring of the polymer onto the MWCNTs, guided by the establishment of directional π–π interactions (Figure 1). Upon sonication with varying amounts of MWCNTs (0–7.5 wt %), **PTPy** was conveniently formulated into a processable ink (**PTPy/MWCNTs**) and directly spray-coated onto flexible polyethylene terephthalate (PET)–indium tin oxide (ITO) electrodes, initiating a long-range self-assembly process driven by the initial recognition and adhesion of the pyrene moiety of **PTPy** onto the MWCNTs, in turn triggering the packing of **PTPy**. The spontaneous process, occurring without activation, yielded a continuous, reproducible nanostructured **PTPy/MWCNT** hybrid electrochromic film characterized by enhanced performance in F-ECDs compared to the bulk **PT**, including faster color switching (*t*_90_^OX^*=* 3.6 s, *t*_90_^RED^*=* 0.3 s; vs *t*_90_^OX^ = 53.2 s, *t*_90_^RED^ = 2.5 s) and improved cyclability (half-life based on Δ*E*, Δ*E*_50%_ = 17.6k cycles vs 278 cycles), all without the need for additional processing steps in the F-ECD production process. Importantly, this method, which can be easily scaled up, can be applied to other ECPs.

## Results and Discussion

### Synthesis of the Pyrene-Derivatized Copolymer **PTPy**

Monomer **1** was synthesized according to published procedures via a *p*-toluenesulfonic acid-catalyzed transesterification reaction of 3,4-dimethoxythiophene with 2-ethylhexanol.^24^**Py**-bearing monomer **2** was prepared by reacting 1-bromomethylpyrene with (2,3-dihydrothieno[3,4-*b*][1,4]dioxin-2-yl)methanol in the presence of NaH in DMF (Scheme S1 and Figures S1–S4). Monomers **1** and **2** were straightforwardly heteropolymerized through a catalyzed oxidative reaction with FeCl_3_,^25^ affording **PTPy** (Scheme S2) as a dark orange, light brown solid. The equivalents of monomer **2** were adjusted (0.1 equiv, 1:10 monomer ratio) to gain sufficient interaction within the whole range of investigated MWCNT loadings (0–7.5 wt %), providing stable dispersions and allowing further processing and device integration.^15^ Monomer **1**-based orange homopolymer **PT** was also prepared as a reference (Scheme S2).

**PTPy** was characterized via ^1^H NMR spectroscopy (Figure S5), UV–vis spectroscopy, and thermogravimetric analysis (TGA; Figure 1c,d). The ^1^H NMR spectrum of **PTPy** displays a broad aromatic feature between 8 and 8.5 ppm, ascribable to the presence of the **Py**-monomer in the backbone of the copolymer (Figure S5), otherwise absent in the ^1^H NMR spectrum of homopolymer **PT** (Figure S6). The UV–vis spectrum of **PTPy** shows a broad absorbance band ranging from 320 to 680 nm with λ_max_ located at 458 nm and with a fingerprint of the pyrene chromophore at ca. 360 nm (Figure 1c). On the contrary, **PT** shows a narrow absorbance (350–590 nm) as compared to **PTPy**. Both **PTPy** and **PT** provided TGA profiles characterized by an intense weight loss between 270 and 400 °C (Figures 1d and S7), corresponding to the pyrolysis of the alkyl side chains. **PTPy**, however, presented a higher residual wt % (i.e., carbonized polymer) at 800 °C compared to **PT** (29.1 and 16.9%, respectively), most likely because of its higher aromatic content (i.e., corresponding to an estimated content of pyrene groups in **PTPy** of ca. 0.5 × 10^–3^ mmol mg^–1^, which roughly translates into a **2**:**1** monomer ratio of 1:6, lower than the 1:10 used for its synthesis).

Due to the scarce solubility of the copolymer, it was not possible to determine its molecular weight by gel permeation chromatography. Thus, the average chain length was determined with a combined electrospray deposition and variable temperature STM analysis performed under ultrahigh vacuum conditions, enabling the visualization of the polymeric strands with monomeric resolution (Figure 1e).^26−28^**PTPy** was electrosprayed from a CHCl_3_/MeOH (4:1 v/v) solution onto a clean Au(111) on a mica substrate under ultrahigh vacuum conditions and analyzed in situ by STM. Figure 1e displays relatively straight, elongated features with sharp bends that are identified as individual polymer strands. The mass distribution of **PTPy** was evaluated by measuring the length of several individual strands, resulting in the distribution shown in Figure 1e (inset), with most of the population lying within the 10–30 monomer window. Despite the absence of strong contrast, a certain regularity could be seen along the polymer backbone with measured periodicities ranging between 0.5 and 0.8 nm. These values are largely consistent with twice the length of a repeat unit along the main polymer axis (0.8 nm), as would be expected for an all-trans conformation of the thiophenyl units. In regions of high local molecular coverage, the polymers assemble parallel to each other with a measured backbone–backbone separation of ∼2.4 nm, indicating that no interdigitation occurs between neighboring side chains.

### Hybridization and Nanostructural Characterization of the **PTPy/MWCNT** Hybrids

**Figure 2:**
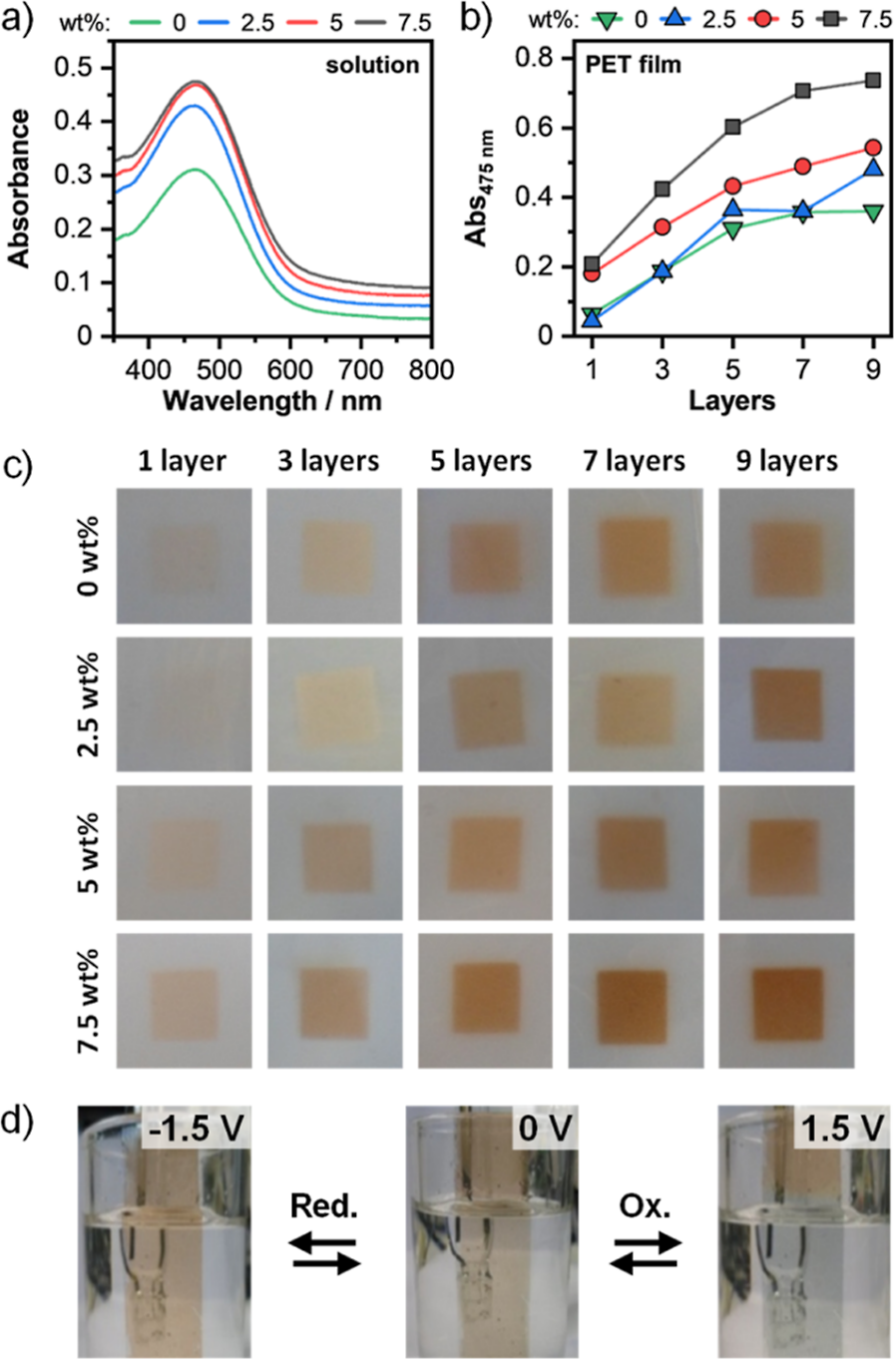
Characterization of the **PTPy/MWCNT** films and solutions. (a) UV–vis absorption spectra of **PTPy** (0.83 mg mL^–1^) blended with MWCNTs (0–7.5 wt %) in CHCl_3_/Tol (5:1 v/v) solutions. (b) Absorbance at 475 nm as a function of the number of layers for the **PTPy/MWCNT** films (1 cm × 1 cm) spray-coated onto PET substrates. (c) Image of PET substrates spray-coated with **PTPy/MWCNT** blends, showing an incremental loading of MWCNTs (0–7.5 wt %) and number of layers (L; 1–9). (d) Electrochromic behavior in solution of **PTPy/MWCNTs** (7.5 wt %) 9 L films spray-coated onto conductive PET–ITO electrodes. Potentials referenced vs Ag/AgCl.

**PTPy/MWCNT** blends containing different amounts of MWCNTs (0–7.5 wt %) were prepared by sonicating **PTPy** (1 mg mL^–1^) in CHCl_3_ for 10 min at 45 °C. The required amount of MWCNTs (0.025–0.075 mg mL^–1^) and toluene (2 mL) were added to the **PTPy** solution. The resulting mixture was further sonicated until a homogeneous dispersion was obtained (see Experimental Section). The concentration of MWCNTs in the stable dispersions was monitored through UV–vis absorption measurements (Figure 2a). The absorbance peak and the typical light brown color of **PTPy** are detectable and unaltered up to 7.5 wt % MWCNT loadings.

Due to its inherent advantages, such as accessibility and versatility, spray-coating was chosen as the preferred manufacturing technology for the preparation of F-ECDs.^29−31^ A preliminary investigation was performed to find the optimal thickness and amount of MWCNTs in the sprayed electrochromic layer (Figure 2). Specifically, freshly prepared **PTPy/MWCNT** inks (0–7.5 wt %) were sprayed onto nonconductive PET sheets (125 μm thick) using an aerograph with a continuously applied pressure (1 bar), yielding **PTPy/MWCNT** films with a different number of layers (Figure 2b,c and Figure S8). The UV–vis absorption spectra of the deposited films showed, as expected, a trend of increasing absorbance upon increasing the loading of MWCNTs, consistent with the observations from the solutions (Figure 2a) and the number of sprayed layers (Figures 2b and S8). The film with 7.5 wt % MWCNTs and 9 spray-coated layers exhibited the strongest absorption (Abs_475nm_ = 0.74) without undesirable darkening caused by the MWCNTs. The optimized film (**PTPy/MWCNTs** 7.5 wt %, 9 layers) was hence sprayed onto a conductive PET–ITO electrode to evaluate its electrochemical switching in solution (Figure 2d). The hybrid film exhibited a color change response when a voltage of +1.5 V was applied, transitioning from a brown hue in the reduced state to a sage green hue in the oxidized state. By reversal of the polarity (i.e., applying −1.5 V), the initial brown color was fully restored. Consequently, these conditions were deemed to be ideal for the manufacturing of F-ECDs.

**Figure 3:**
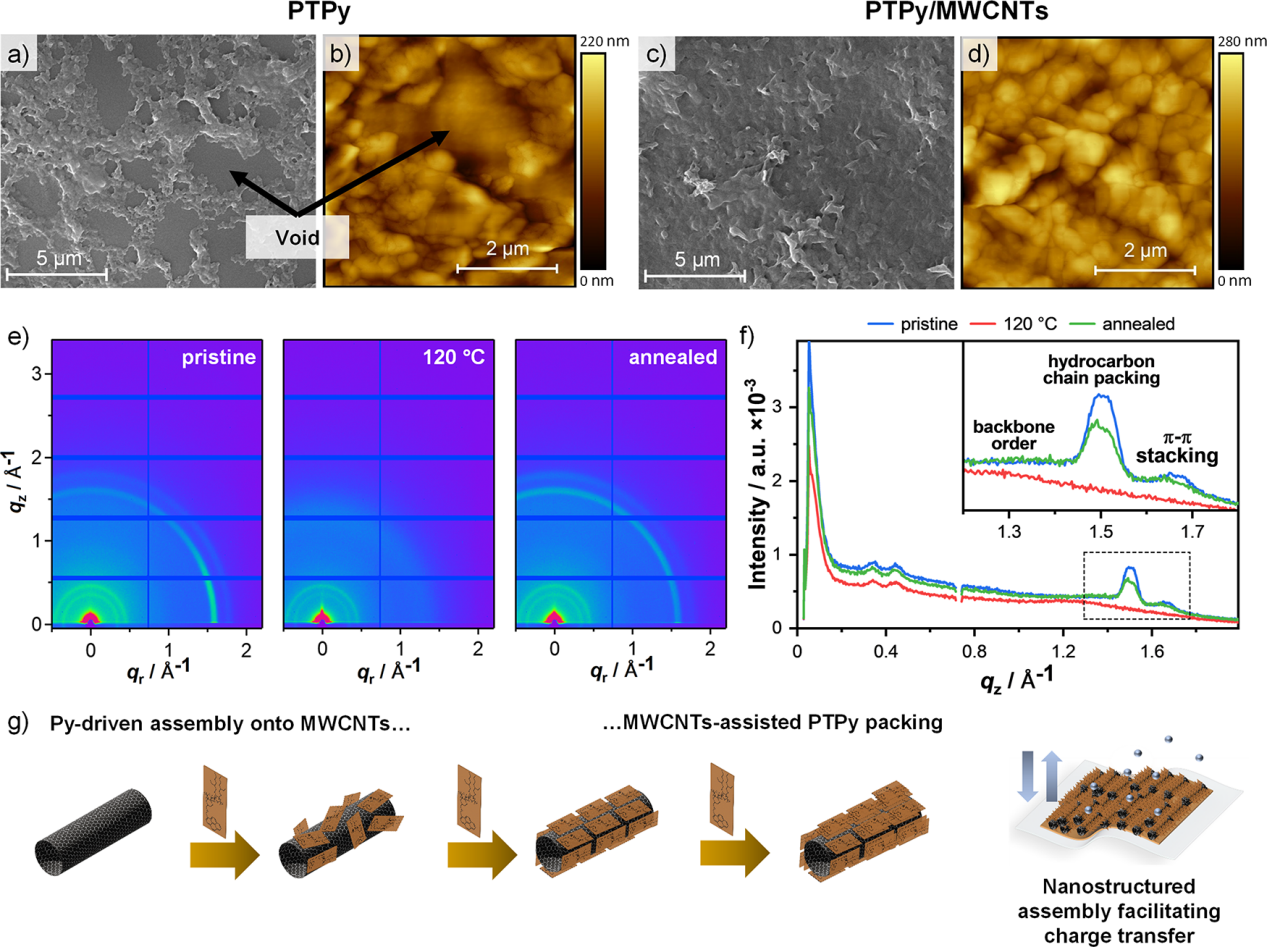
Morphological characterization of **PTPy/MWCNT** films. SEM and AFM images of (a,b) **PTPy** and (c,d) **PTPy/MWCNT** (7.5 wt %) spray-coated onto a silicon substrate. (e) 2D-GIWAXS images of **PTPy/MWCNT** (7.5 wt %) spray-coated on a silicon chip at rt, upon heating to 120 °C (2 °C min^–1^), and subsequent cooling back to rt. (f) Radially integrated intensity profile of the corresponding GIWAXS images. (g) Graphical representation of the pyrene-driven assembly process.

To elucidate the morphology and nanostructure of the hybrid electrochromic films, we performed microscopic and X-ray scattering analyses of **PTPy/MWCNTs** (7.5 wt %). For analysis, samples were spray-coated onto a silicon chip, employing the same procedure optimized for device preparation (**PTPy** films were also prepared for comparison purposes). We first investigated the morphology of **PTPy/MWCNT** films by scanning electron microscopy (SEM) and atomic force microscopy (AFM). The top-view SEM images of **PTPy** and **PTPy/MWCNT** films reveal distinct film morphologies depending on whether the copolymer is deposited alone or as a hybrid (Figures 3a,c, S9, and S10). Specifically, **PTPy** films exhibit a nonhomogeneous and porous structure consisting of polymeric aggregates (composed of particles in the range of 200–300 nm), which do not exhibit continuity among them (Figure 3a). In contrast, **PTPy/MWCNT** hybrids reveal a continuous, uniform film that embeds aggregates throughout the entire sample (Figure 3c). At higher magnification, the particulate morphology observed in the **PTPy** sample is absent and replaced by a homogeneous, continuous, and rough coating in the hybrid film. These observations are consistent with the AFM measurements (Figures 3b,d and S11).

As the presence of MWCNTs could not be easily detected in the **PTPy/MWCNT** films, energy-dispersive X-ray (EDX) spectroscopy analysis was also performed. This analysis revealed a higher average C atomic percentage of 59.8 at % in the **PTPy/MWCNT** films, compared to 47.6 at % in the **PTPy** film (Table 1), affirming the incorporation of MWCNTs into the hybrid film. Knowing that soluble polythiophenes composed of a rigid conjugated backbone and flexible side chains (e.g., P3HT) have the potential to generate crystalline films through π–π interactions,^32^ we investigated the nanostructuring of the hybrid film via grazing-incidence wide-angle X-ray scattering (GIWAXS) analysis both under annealed and nonannealed conditions (Figures 3e,f, S12–S17, and Table S1).

**Table 1:** Elemental Analysis Performed via EDX Analysis, Reporting the Atomic Percentages of the Different Elements Detected in the **PTPy** and **PTPy/MWCNT** (7.5 wt %) Samples

MWCNTs (wt %)	C (at %)^a^	In (at %)^a^	O (at %)^a^	Sn (at %)^a^	S (at %)^a^
0	47.6 ± 0.3	27.7 ± 0.2	21.5 ± 0.1	1.9 ± 0.1	1.3 ± 0.1
7.5	59.8 ± 0.4	23.0 ± 0.4	14.3 ± 0.1	2.2 ± 0.1	0.7 ± 0.1

a Average of data collected on three different spots.

Analysis of the 2D GIWAXS diffraction pattern of the as-prepared **PTPy/MWCNT** film revealed two sharp diffraction peaks at scattering vectors *q* = 1.5 and 1.66 Å^–1^ and a broad peak at 1.4 Å^–1^. The first two reflections with corresponding *d*-spacings of 4.2 and 3.8 Å are ascribable to the hydrocarbon chain packing and π–π stacking interactions of **PTPy** induced by its attachment onto the **MWCNTs**. The broad reflection (*d*-spacing of 4.5 Å) with a coherent domain size of approximately 4 nm corresponds well to the order along the backbone axis of the polymer (Table S1), indicating dense packing of polymer chains that could facilitate charge hopping within the film. The additional reflections around 0.4 Å^–1^ arise from the Kapton windows of the heating cell. This data indicates long-range order and π–π stacking corresponding to the presence of nontextured **PTPy** crystallites isotropically oriented in the film before annealing. Temperature-dependent studies showed that at temperatures >110 °C, the sharp diffraction rings disappear, indicating loss of crystallinity (Figures 3e,f and S12–S15). Only the backbone and a broad correlation peak between hydrocarbon chains/π–π stacking pertains to the original order (Figures S12–S15). Upon cooling, the features reappear, suggesting reversibility and efficiency for the π–π interactions in bringing the two materials together, although accompanied by a minimal loss of intensity (Figures 3e,f and S16). As recently reported by Stefan and co-workers for assembled films of **Py**-functionalized P3HT,^33^ annealing caused an apparent loss in signal intensity ascribable to the formation of highly oriented crystals with lamellar stacking strongly textured in the out-of-plane direction, which GIWAXS data cannot fully capture. Notably, GIWAXS analysis of **PT/MWCNT** hybrids (Figure S17) did not afford any scattering vectors at *q* = 1.50 and 1.64 Å^–1^ indicating the absence of any crystallinity and hence adsorption of the polymer onto the MWCNTs. As proposed by Lazzaroni and co-workers, **Py**-containing P3HT oligomers can tangentially adsorb on a CNT surface through a staggered stacking mediated by the pyrenyl functionalities.^34^ The presence of the terminal **Py** unit was found to enhance the adsorption energy by about 20% for a Py-(3HT)_10_ chain compared to the underivatized polymer. Building on these results, we suggest that the dangling pyridyl moieties in **PTPy** establish stabilizing π–π interactions with the graphitic NT surface, triggering the self-assembly of the polymeric units onto the MWCNTs (Figure 3g). On the other hand, the absence of the polycyclic aromatic structures and the presence of bulkier substituents (i.e., a set of 2-ethyloxhexyloxy substituents per thiophene vs one hexyl chain per thiophene unit in P3HT) in **PT** hamper the interaction with MWCNTs. It is thought that initially **PTPy** adsorbs stochastically, and as the surface saturates, the **PTPy** chains begin to interact, adjusting to find an energetically favorable assembly before initiating the adsorption of another layer. Eventually, this process leads to the formation of a crystalline microstructure responsible for the enhanced charge mobility of the hybrid (Figure 3g).

### Prototyping F-ECDs, Electrochromic Performance, and Manufacturing of a Large-Scale Electrochromic Device

**Figure 4:**
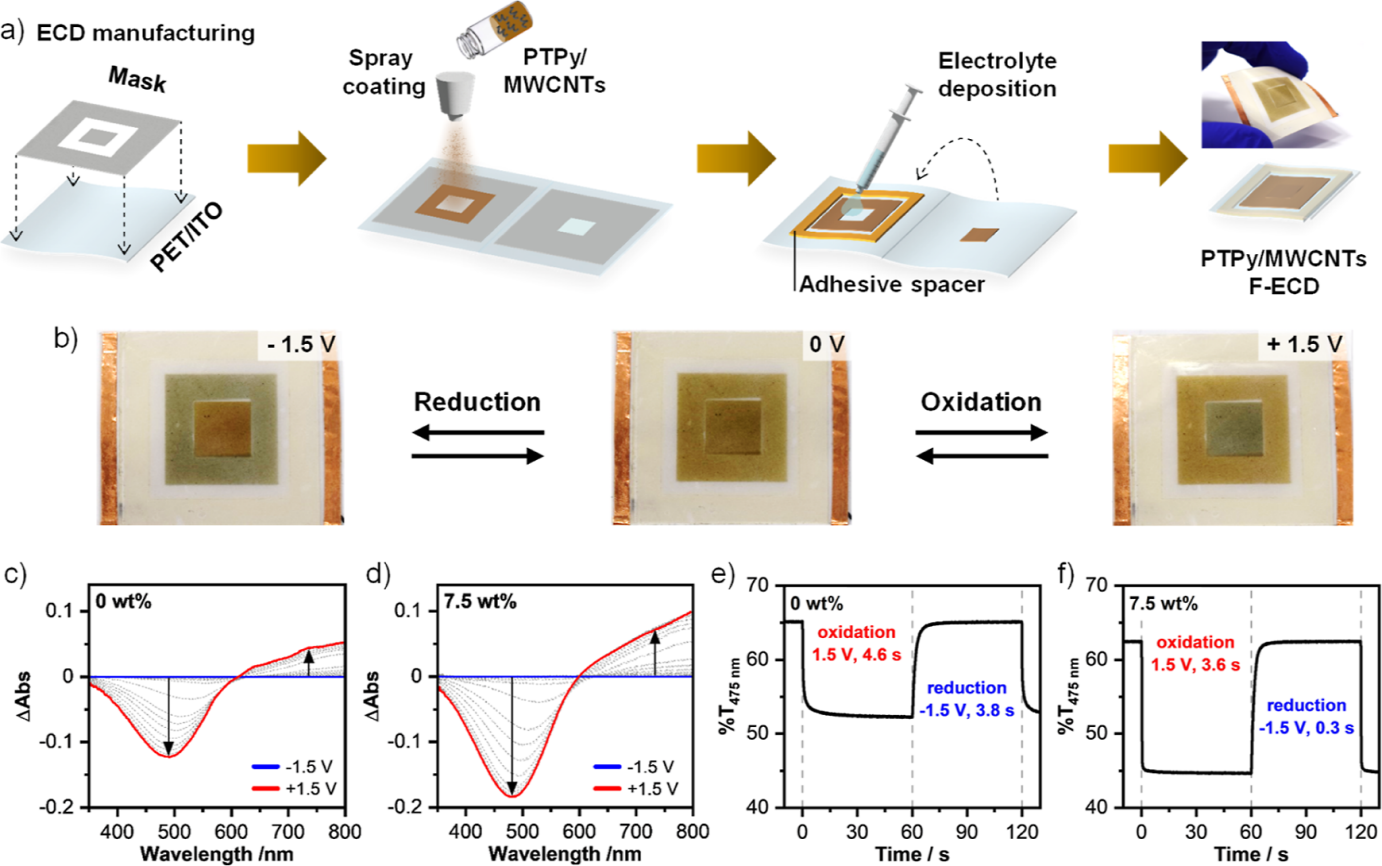
Evaluation of the electrochromic behavior of **PTPy/MWCNT** F-ECDs. (a) Graphical representation illustrating the manufacturing process of F-ECDs. (b) Images of a **PTPy/MWCNT** (7.5 wt %) F-ECD taken upon application of 0 and ∓1.5 V. (c,d) Change in absorbance (350–800 nm) of **PTPy** and **PTPy/MWCNT** (7.5 wt %) F-ECDs upon electrochemical switching from −1.5 to +1.5 V. (e,f) Electrochemical switching monitored at 475 nm upon voltage cycling between −1.5 and +1.5 V.

Solid-state F-ECDs were assembled by following a vertical stack architecture. This involved the deposition of an UV curable Li^+^-based gel electrolyte layer^35^ between the previously sprayed PET–ITO electrodes, which were then laminated using a double-sided pressure-sensitive adhesive tape (Figure 4a). For device characterization, a square device design comprising an active area of 1 cm^2^ was adopted (Figures S18 and S31).

The spectroscopic variation (i.e., UV–vis absorption) of the bias-induced color switching in **PTPy/MWCNT** F-ECDs was investigated by performing in situ spectroelectrochemical measurements (Figures 4c,d and S19). Upon application of an increasingly higher voltage bias, all devices exhibited the brown-to-green reversible color transition. The maximum difference in absorbance between the reduced and oxidized states (ΔAbs) is observed in the visible region of the UV–vis spectrum at 475 nm. This corresponds to a decrease in the π–π* transition of the neutral polymer (Figure 1c).

At the same time, electronic transitions in the red/NIR regions become apparent in the oxidized polymer. This phenomenon has been previously described by Heeger^36^ and others,^37−39^ and is attributed to an enhancement of conjugation in the polymer backbone upon the formation of bipolarons during oxidation. This enhanced conjugation is responsible for the dramatic color change observed during the electrochemical switching. The spectroelectrochemical profile measured for the devices without MWCNTs exhibited a very similar shape (Figure S19), suggesting the presence of MWCNTs has a negligible impact on the coloration of the film.

Spectro chronoamperometric measurements were conducted to determine relevant F-ECD parameters (Figures 4e,f and S20, and Table 2), namely: the oxidation and reduction switching time (*t*_90_^OX^, *t*_90_^RED^), the maximum transmittance variation (Δ%*T*), and oxidation and reduction coloration efficiency (η^OX^, η^RED^) values. The coloration efficiency parameter is defined as the change in optical density (ΔOD) at a specific wavelength of the material per unit of charge (Δ*Q*) intercalated into or extracted from the EC film.^40^ This relationship is expressed by the equation
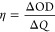
where ΔOD is calculated as ΔOD = log(*T*_ox_/*T*_red_), and Δ*Q* is calculated by integrating the current curve over time. This parameter provides a measure of how efficiently the material modulates its optical properties in response to the applied charge. A higher η value indicates that a smaller amount of charge is needed to achieve a significant optical change, which is crucial for applications in which energy efficiency and rapid switching are important. Similarly to the switching time, we calculated the coloration efficiency for both reduction and oxidation processes and determined the values corresponding to a 90% change of the total ΔOD (Figure S21). During the measurements, the transmittance at 475 nm was recorded while simultaneously applying a square wave with a peak-to-peak amplitude of 3 V (−1.5 to +1.5 V) and a period of 120 s.

**Table 2:** Electrochromic Data of **PTPy/MWCNT** Films as a Function of the MWCNT Percentage

MWCNTs (wt %)	Δ%*T*	Δ*E*	*t*_90_^OX^ (s)	*t*_90_^RED^ (s)	*Q*_90_^OX^ (mC cm^–2^)	*Q*_90_^RED^ (mC cm^–2^)	η_90_^OX^ (cm^2^ C^–1^)	η_90_^RED^ (cm^2^ C^–1^)
0	13	13.7	4.6	3.8	1.10	0.95	81.1	91.7
7.5	17.8	24.5	3.6	0.3	1.22	0.58	107.9	225.4

While the spectroelectrochemical profiles measured for the devices with and without MWCNTs exhibit similar patterns (Figure S19), the switching time of the F-ECDs yielded strikingly different results (Figure 4e,f). Specifically, F-ECDs containing the MWCNT-based hybrid showed a significant decrease in the reduction switching time (*t*_90_^RED^, Table 2), which decreased from 3.8 s for the **PTPy**-only device to 0.3 s. This corresponds to a 92% decrease and results in more than 10 times faster switching times. The oxidation switching time (*t*_90_^OX^, Table 2) also demonstrates a decrease, although with a lesser magnitude compared to the reduction switching time (approximately 22% faster). Along with the improvements observed for the switching times, we could also observe an increase in the η values in the presence of MWCNTs (Table 2). To corroborate the critical role of the pyrene moieties in structuring the interaction of **PTPy** with MWCNTs, a reference experiment was performed using a blend of the pyrene-free homopolymer analogue, **PT**, with 7.5 wt % MWCNTs (Figures S22–S24 and Table S2). Since this polymer was found to be almost incompatible with MWCNTs, sonication was prolonged to 6 h to achieve a sprayable ink. Compared to **PTPy**, the pristine **PT** presents similar *t*_90_^RED^ (2.5 s; Table S2), yet a significantly higher *t*_90_^OX^ (53.2 s) renders the overall electrochemical switching process highly inefficient. Upon blending with 7.5 wt % MWCNTs, the resulting **PT/MWCNT** hybrid displayed a decrease in both oxidation (25.8 s) and reduction switching times (1.7 s). However, the oxidation switching time remains excessively high compared with the reduction switching time. The addition of MWCNTs also does not particularly affect the coloration efficiency of the hybrid (Table S2).

**Figure 5:**
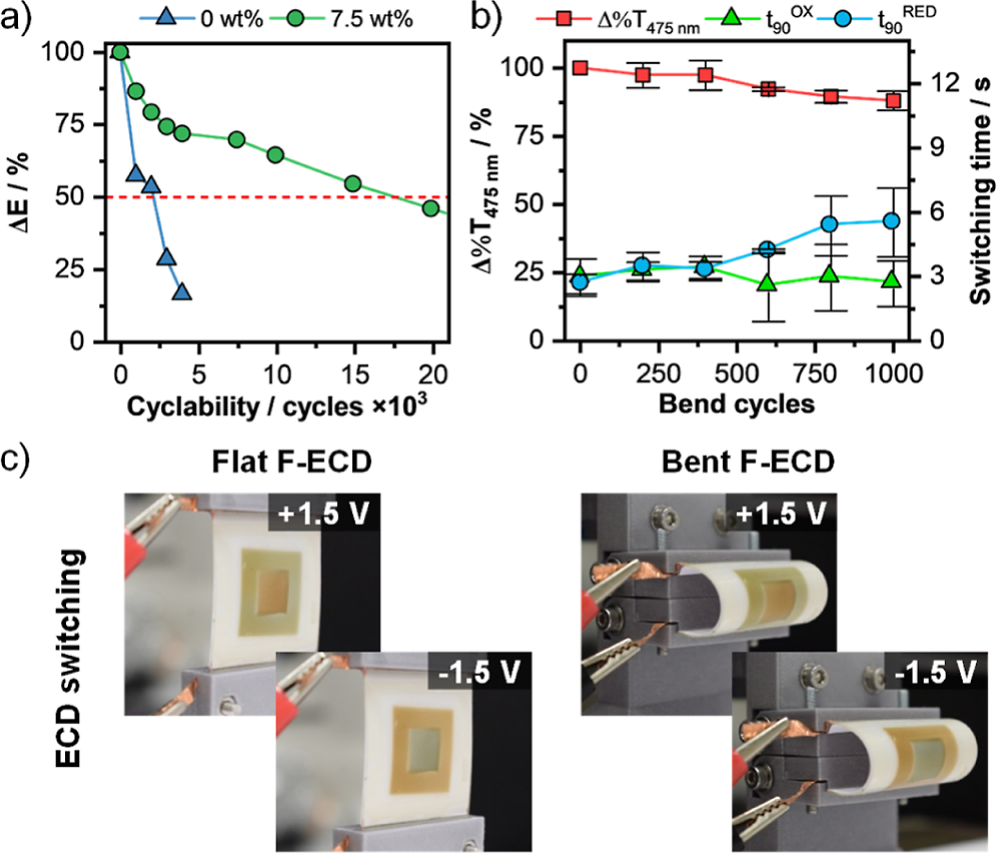
Evaluation of **PTPy/MWCNT** F-ECDs stability under electrochemical and mechanical stress. (a) Color contrast monitored as a function of the number of electrochemical cycles (±1.5 V, 5 s) for devices assembled with **PTPy** and **PTPy/MWCNTs** (7.5 wt %). (b) Transmittance change (at 475 nm) and switching times during electrochemical switching monitored as a function of the number of physical bends (bend radius = 10 mm) for **PTPy/MWCNT** (7.5 wt %) F-ECDs. (c) Images of **PTPy/MWCNT** (7.5 wt %) F-ECDs switching before and during bending.

Finally, we investigated the stability of the F-ECDs under continuous electrochemical switching by monitoring the color difference (Δ*E*, see Supporting Information) value, which represents the total color contrast over time. The stability was evaluated in terms of the half-life, defined as the number of cycles required for a 50% loss of starting color contrast (Figures 5a and S25–S28). The plots clearly show that the addition of MWCNTs significantly increases the cycling stability of the devices (Figure 5a). The polymer-based device (i.e., 0 wt % MWCNTs) reached its half-life after only 2.2k cycles, indicating relatively poor stability. However, the device containing 7.5 wt % MWCNTs exhibited a drastic improvement in stability, lasting for up to 17.6k cycles. We also calculated the reduction and oxidation switching times over a number of cycles, with both values being below 2 s and no significant variation observed (Figure S29). In comparison, both **PT** and **PT/MWCNT** F-ECDs lost functionality after only 278 and 282 cycles, respectively (Figure S30). This highlights the superior stability achieved by nanostructuring the incorporation of MWCNTs into the hybrid electrochromic layer. It is important to note that the high durability achieved for the **PTPy/MWCNT** device surpasses the values reported for state-of-the-art organic and hybrid F-ECDs (Table S3).

Bendability tests were performed to demonstrate the resilience of the F-ECDs to mechanical stress (Figures 5b,c and S31, and Movies S1, S2, and S3). The devices were bent up to 1k times with a radius of 10 mm and subjected to a series of bending cycles at a rate of 4 cycles min^–1^. The bending was kept blunt to avoid detrimental cracking of the conductive ITO layer.^41,42^ The electrochromic performance (i.e., Δ%*T* and *t*_90_) was monitored as a function of the bending cycles while the bending was ongoing. As shown in Figure 5b, the color contrast and switching time values of the device were not significantly affected upon bending. For example, the Δ%*T* values before and after 1k cycles were 16.7 and 14.7%, respectively, indicating the excellent resilience to mechanical stress of the **PTPy/MWCNT** (7.5 wt %) films.

**Figure 6:**
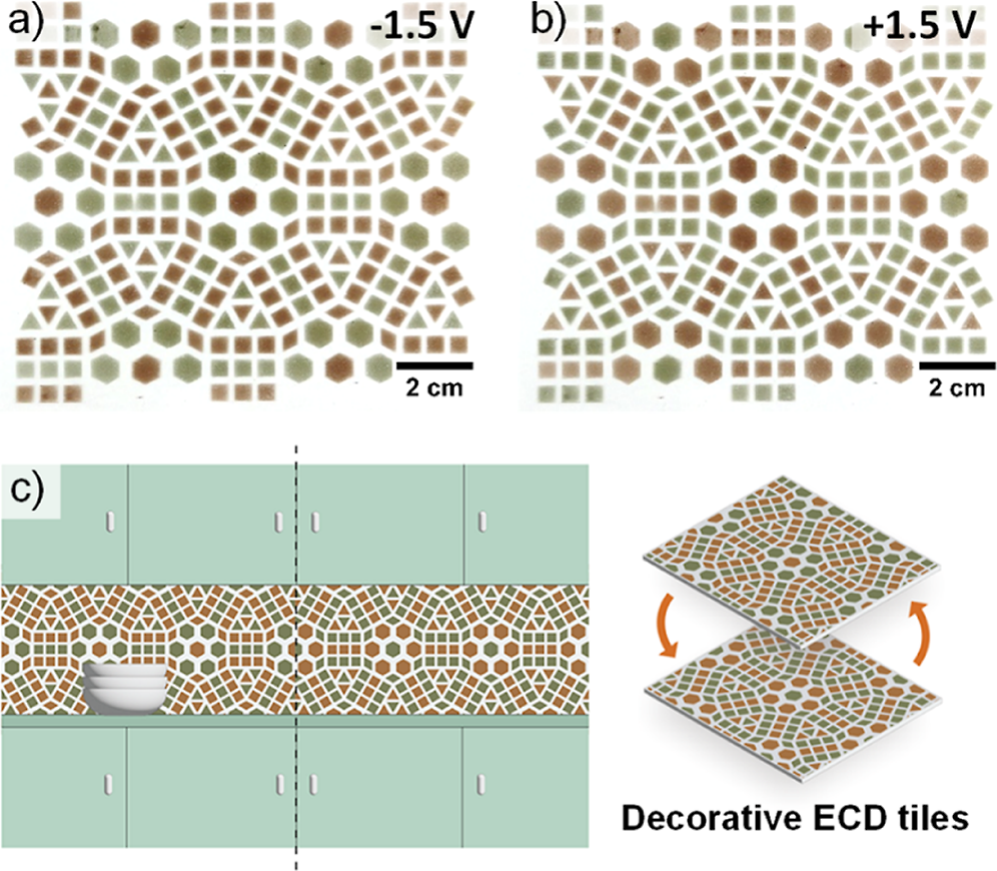
(a,b) Image of a large area (11 × 13 cm) **PTPy/MWCNT** (7.5 wt %) F-ECD in its reduced (a) and oxidized state (b). (c) Example of the potential application of large-scale F-ECDs as decorative color-changing tiles, eventually responding to a stimulus as imagined for an IoT-integrated system.

To further exploit the performance enhancement achieved by blending **PTPy** with MWCNTs, a large-area F-ECD (11 × 13 cm, total active area ∼20 cm^2^) was manufactured using the same spray-coating procedure employed for the small-scale (1 cm^2^) devices (Figure 6a,b), featuring an azulejos-type tile pattern (Figure S32).

Typically, electrochromic devices of such large sizes suffer from a slow switching time and reduced cyclability. Surprisingly, even at this scale, the F-ECD displayed fast switching in both oxidation (*t*_90_^OX^ = 0.8 s) and reduction (*t*_90_^RED^ = 0.9 s) processes, with a color contrast of 9.5 Δ*E* (Figures 6a,b and S33). These values are comparable to F-ECDs with surface areas of 1 cm^2^, demonstrating the remarkable performance of a large-area F-ECD despite its size.

The expanded active area of our efficient, long-cycling **PTPy/MWCNT** F-ECDs offers numerous possibilities for applications, including decorative and interactive features in an IoT smart home setting. For instance, decorative interactive tiles embedded with this technology (Figure 6c) could provide a stimulating and pleasant effect by allowing users to trigger a change in color patterns. Alternatively, the color change of these tiles could be associated with monitoring and controlling various aspects of the smart home, such as lighting, temperature, and appliances. The interactive F-ECD would then not only enhance convenience but also empower users with comprehensive insights and customization options, making living spaces more aesthetically pleasing, intuitive, and responsive to the users’ needs.

## Conclusions

In summary, we successfully developed a template-induced supramolecular assembly approach, leveraging MWCNTs as templates for fabricating nanostructured hybrid pyrene-derivatized ECP/MWCNT (i.e., **PTPy/MWCNT**) electrochromic films. These were characterized by an engineered and reproducible nanostructuring, producing a continuous film that could allow better improving charge and discharge processes and stability in operating conditions, improving the performances of the bulk ECP. The hybrid F-ECDs, produced via a streamlined spray-casting process enabling scalability, exhibited improved color switching (*t*_90_^OX^ = 3.6 s, *t*_90_^RED^ = 0.3 s) and a steep increase in cyclability (Δ*E*_50%_ = 17.6k cycles) compared to the unstructured **PTPy** (*t*_90_^OX^ = 4.6 s, *t*_90_^RED^ = 3.8 s; Δ*E*_50%_ = 2.2k cycles) and the initial **PT** polymer (*t*_90_^OX^ = 53.2 s, *t*_90_^RED^ = 2.5 s; Δ*E*_50%_ = 278 cycles), even under mechanical stress. Notably, the same improvements were recorded also for larger-scale devices (20 cm^2^; *t*_90_^OX^ = 0.8 s, *t*_90_^RED^ = 0.9 s), uncharacteristic for such sizes. This approach, which can be extended to all the polythiophene-type ECPs, offers significant potential for commercializing multicolored F-ECDs by leveraging the readily available, diverse color palette of ECPs, compatibility with existing manufacturing processes, simplicity, and scalability. Additionally, the ability to prototype larger, high-performance F-ECDs opens new possibilities for IoT applications, particularly in responsive and decorative displays.

## Experimental Section

### Instrumentation

Thin-layer chromatography was conducted on precoated aluminum sheets with 0.20 mm Merck Millipore silica gel 60 with fluorescent indicator F254. Column chromatography was carried out using silica gel 60 (230–400 mesh, Merck). All ^1^H and ^13^C NMR spectra were recorded using a Bruker AV-400, AV-600 spectrometer, or AV-700 spectrometer at 300 K. Chemical shifts were reported in ppm according to tetramethylsilane using the solvent residual signal as an internal reference (CDCl_3_: δ_H_ = 7.26 ppm, δ_C_ = 77.16 ppm). Coupling constants (*J*) are given in Hz. Resonance multiplicity was described as s (singlet), d (doublet), dd (doublet of doublets), m (multiplet), and br (broad signal). Carbon spectra were acquired with complete decoupling for the proton. Mass spectra were obtained using a Finnigan MAT 8200 (70 eV) or an Agilent 5973 (70 eV) spectrometer. UV–vis absorption spectroscopy was performed on a Cary 5000 UV–vis–NIR spectrophotometer (Agilent, US). For spectroelectrochemical measurements, the F-ECDs were connected to a AutoLab PGSTAT 100N potentiostat (Metrohm, CH), and continuous voltage was applied (at selected values) for 60 s, after which the absorbance spectra were recorded. For switching time measurements, a pretreatment of 15 cycles (−1.5/1.5 V, 10 s period) was applied, followed by three cycles with a 120 s period, while monitoring the % *T* change at a fixed wavelength. Chronoamperometry measurements were performed with a pretreatment of 15 cycles (10 s period), followed by 3 cycles with a 120 s period. The charges consumed by the F-ECDs during a cycle were calculated from the current developed during the experiment through the integration of the chronoamperometric signal. All TGAs were performed with a TGA 550 (TA Instruments, US) under a N_2_ flow of 60 mL min^–1^ and with the following method: equilibration from room temperature to 100 °C, isothermal heating at 100 °C for 30 min, then ramp from 100 to 800 °C (heating rate of 10 °C min^–1^). SEM images were recorded with a Zeiss Supra 55 VP instrument (Carl Zeiss, DE) with an acceleration voltage of 5 kV. The sample was prepared by spraying the **PTPy/MWCNT** dispersion onto a Si substrate (1 cm^2^), subsequently sputter coated with Au (Emitech K575X Peltier cooled) for 60 s at 60 mA prior to fixation on an Al support. EDX spectroscopy mapping was performed at an acceleration voltage of 20 kV. AFM investigations were performed using a Nanoscope V MultiMode 8 instrument (Veeco, US). The measurement was performed in tapping mode at room temperature with a POINTPROBE-PLUS Silicon-DPM-Sensor cantilever. The hybrid materials were sampled by acquiring 3 images per sample in different areas (5 × 5 μm, 90 min acquisition, scan rate 0.2 Hz). The Gwyddion^43^ software was used to analyze the images. GIWAXS patterns have been recorded at the Austrian SAXS beamline at ELETTRA^44^ with a Pilatus3 1M detector at a sample-to-detector distance of 206 mm using an X-ray energy of 8 keV. The spot size at the sample position has been set to 0.2 × 1.5 mm (vertical × horizontal). The data were corrected for fluctuations in the primary intensity. GIXRD patterns were taken at a grazing angle of 0.2° (below the critical angle of Si). The horizontal cuts at the Yoneda wing have been performed with SAXSDOG,^45^ and the cuts have been analyzed with IGOR Pro (WaveMetrics, US) using the Multi-peak fitting tool with a third order polynomial as background. STM experiments were performed under ultrahigh vacuum in a SPM Aarhus 150 (SPECS, DE) variable-temperature STM system. Images were acquired at −143 °C in constant-current feedback mode, with typical sample bias between −2 and −1.5 V and tunneling currents of 30–50 pA. The Gwyddion^43^ and LMAPper^46^ software were used to analyze the images. Samples were electrosprayed (Molecularspray Ltd., UK) from a CHCl_3_/MeOH (4:1 v/v) solution under ultrahigh vacuum conditions onto 300 nm Au(111)/mica substrates (Georg Albert PVD, DE), which were previously cleaned by Ar^+^ ion sputtering followed by annealing.

### Materials

Chemicals were purchased from Sigma-Aldrich, TCI, and ABCR and were used as received. Solvents were purchased from Sigma-Aldrich, and deuterated solvents were from Eurisotop. Solvents for spectrophotometry were purchased from Acros Organics and Jansen Chemicals. MWCNTs (NC7000) were purchased from Nanocyl.

### Preparation of the **PTPy/MWCNT** Blends

Ten mg of **PTPy** and 10 mL of CHCl_3_ (1 mg mL^–1^) were added to a glass vial, and the obtained dispersion was sonicated for 10 min at 45 °C (150 W, 37 kHz). Afterward, the desired amount of MWCNTs (0.025–0.075 mg) was added as a solid, followed by 2 mL of toluene. The obtained mixture was once again sonicated for 30 min at 45 °C, producing a homogeneous and stable dispersion.

### Spray-Coating of **PTPy/MWCNT** Hybrids

The spray-casting of the blends on the PET–ITO substrates was performed by using an aerograph with a continuously applied pressure of 1 bar for each layer. To ensure the homogeneity of the depositions, the aerograph with a 0.3 mm opening was kept at a constant distance from the substrate (5 cm) so that only the aerosol part of the sprayed mixture was deposited into the plastic substrate. Additionally, the spraying rate of the produced blends was kept constant for each layer and performed on PET–ITO placed on top of a heating plate kept at 60 °C to promote fast evaporation of the solvent.

### Manufacturing of F-ECD Devices

Solid-state F-ECDs were assembled using a vertical stack architecture by depositing a UV-curable Li^+^-based gel electrolyte1 layer between the previously sprayed PET–ITO electrodes and laminating them together with a double-sided pressure-sensitive adhesive tape. The same procedure applies to the preparation of the large-area F-ECD.

### Solution Switching of **PTPy/MWCNT** Films

To evaluate the color switching of the **PTPy/MWCNT** blends, spray-coated PET–ITO electrodes were placed in an electrochemical cell with a three-electrode configuration comprising a platinum wire as a counter-electrode and a Ag/AgCl electrode as a reference. 0.1 M LiClO_4_ in propylene carbonate was used as a supporting electrolyte. Potentials between −1.5 and +1.5 V were applied to the working electrode to trigger the color change.

### Cycling Experiments

A camera (IDS, DE), a diffuse lamp (ML series Cold-Cathode Light Panel from Vision Light Tech, NL), and a ColorChecker classic (X-Rite, US) were used to set up a cycling chamber. The ECDs were placed inside the chamber and connected to a function generator programmed to apply a 1.5 V/–1.5 V square wave with a period of 60 s. The camera was set up to record 150 pictures (1 picture/s) in regular intervals, until the devices stopped working or displayed negligible switching. The pictures were then linearized using ColorChecker and converted to L*a*b* color coordinates using a custom MATLAB script.

### Bendability Tests

The F-ECD bending tests were performed by using a custom-built bending machine based on a 3D printer (Prusa Research, CZ). The F-ECDs were clamped in place between two movable stages that linearly moved closer together until a bend radius of 10 mm was achieved.

## Data Availability

All procedures for the synthesis are reported in the Supporting Information.

## Supporting Information Available

Synthetic procedures, NMR spectra, and photophysical and electrochemical characterizations (PDF)
Demonstrating of ECD switching while bending (AVI)
Demonstrating of ECD switching while bent (AVI)
Demonstrating of ECD switching while flat (AVI)

## Supplementary Material

em4c00534_si_001.pdf

em4c00534_si_002.avi

em4c00534_si_003.avi

em4c00534_si_004.avi
