# Supplementary material for: Long-Range Supramolecular Assembly of a Pyrene-Derivatized Polythiophene/MWCNT Hybrid for Resilient Flexible Electrochromic Displays
Source: ACS Appl Eng Mater. 2024 Oct 21;2(11):2640–50. doi: 10.1021/acsaenm.4c00534 (PMC11590053; doi:10.1021/acsaenm.4c00534)
Supplement: Supplementary file 1 — em4c00534_si_001.pdf [file em4c00534_si_001.pdf]

Supporting Information

Long-range Supramolecular Assembly of a Pyrene-derivatized Polythiophene/MWCNTs Hybrid for Resilient Flexible Electrochromic Displays

Rúben R. Ferreira,‡[a] *Dario Mosca,*‡[b] *Tiago Moreira,*‡[c] *Vivek Chandrakant Wakchaure,*[a] *Gianvito Romano,*[a] *Antoine Stopin,*[a,d] *Carlos Pinheiro,*[e] *Alexander M. T. Luci,*[f] *Luís M. A. Perdigão,*[f] *Giovanni Costantini,*[g] *Heinz Amenitsch,*[h] *Cesar A.T. Laia,*[c] *A. Jorge Parola,*[c] *Laura Maggini**[a] and *Davide Bonifazi**[a,d]

[a]Institute of Organic Chemistry, University of Vienna, Währinger Straße 38, 1090 Vienna, Austria.
E-mail: davide.bonifazi@univie.ac.at; laura.maggini@univie.ac.at

[b]Department of Chemistry and Namur Research (NARC), University of Namur (UNamur), Rue de Bruxelles 61, 5000 Namur, Belgium.

[c]Department of Chemistry, Faculty of Science and Technology, Universidade NOVA de Lisboa. Campus de Caparica, 2829-516 Caparica, Portugal.

[d]School of Chemistry, Cardiff University, Park Place Main Building, Cardiff CF10 3AT, United Kingdom.

[e]Ynvisible GmbH, Engesserstr. 4A 79108 Freiburg, Germany.

[f]Department of Chemistry, University of Warwick, Gibbet Hill Road, Coventry, CV4 7AL United Kingdom.

[g]School of Chemistry, University of Birmingham, Birmingham B15 2TT, United Kingdom

[h]University of Technology, Institute for Inorganic Chemistry, Stremayergasse 9/V, 8010 Graz, Austria.

Table of contents

1. Synthetic procedures 3
2. NMR spectra 7
3. Supporting figures and tables 10
4. References 28

1. Synthetic procedures

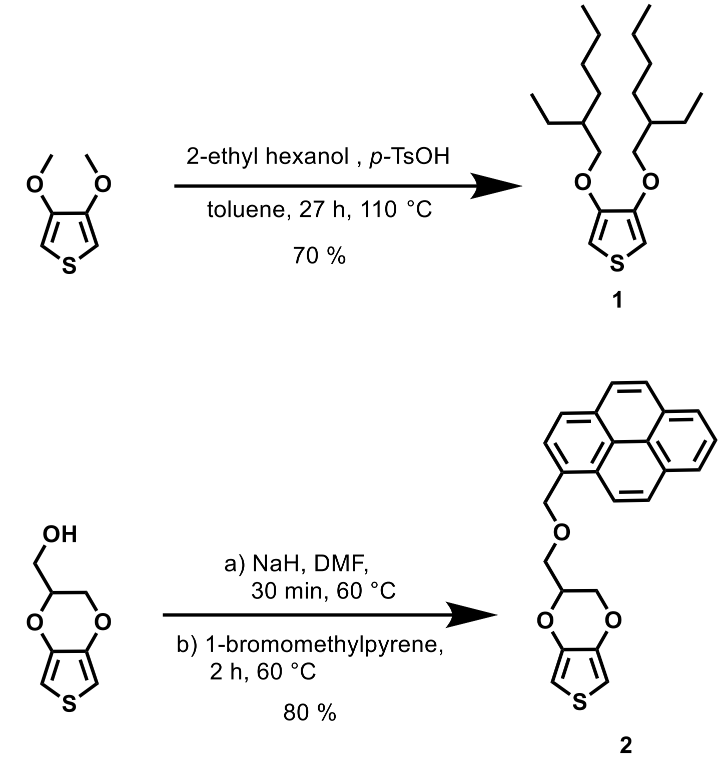

Scheme S1. Synthetic scheme of monomers **1** and **2**.

Synthesis of monomer 1. A solution of 3,4-dimethoxythiophene (200 mg, 1.39 mmol), 2-ethylhexanol (0.92 mL, 5.88 mmol), and *p*-toluenesulfonyl acid (31 mg, 0.163 mmol) in dry toluene was stirred for 27 hours at 110 °C. During this time, the methanol formed during the reaction was regularly released from the flask through a needle. After cooling to rt, H_2O (10 mL) was added, and the phases were separated. The organic phase was washed with H_2O (2×10 mL), dried over Na_2SO_4, filtered and the solvent removed under reduced pressure. The crude material was purified by silica gel chromatography (Silica Gel 60, 0.04-0.06 mm; eluent: Hexane/DCM: 100/0 to 80/20) to yield the desired compound as a light-yellow oil (331 mg, 70 % yield).

^{1}H NMR (300 MHz, $CDCl_3$) δ (ppm) 6.17 (*s*, 2H), 3.85 (*d*, 4H, J = 5.9 Hz), 1.76 (*m*, 2H), 1.65-1.19 (*m*, 16H), 0.92 (*m*, 12H).

^{13}C NMR (75 MHz, $CDCl_3$) δ (ppm) 148.2, 97.0, 73.3, 73.3, 39.4, 30.8, 29.2, 24.1, 23.2, 14.2, 11.3. (One carbon signal more than the expected number due to the presence of diastereomers). Characterization in agreement with reported literature.[1]

Synthesis of monomer 2. A dispersion of NaH (60% in mineral oil, 390 mg, 9.75 mmol) and 2,3-dihydrothieno[3,4-b]-1,4-dioxin-2-methanol (961 mg, 5.58 mmol) in anhydrous DMF (20 mL) under argon was heated at 60 °C for 30 minutes, and 1-bromomethylpyrene (2.021 g, 6.85 mmol) was added, the mixture was stirred for 2 hours at 60 °C. Afterward, the solvent was evaporated in vacuo and the crude was purified by silica gel column chromatography (eluent: n-hexane/AcOEt 8:1), affording a yellow powder. Finally, the powder was solubilized in CH_2Cl_2 and precipitated using MeOH affording the desired compound as a yellow-greenish powder (1.73 g, 80% yield).

^{1}H NMR (300 MHz, $CDCl_3$) *δ* (ppm) 8.37 (*d*, 1H, *J* = 9.2 Hz), 8.21-8.02 (*m*, 8H), 6.35 (*d*, 2H, *J* = 3.6 Hz), 6.32 (*d*, 2H, *J* = 3.6 Hz), 5.35 (*m*, 2H), 4.38-4.32 (*m*, 1H), 4.18 (*m*, 1H), 4.08-4.03 (*m*, 1H), 3.82-3.76 (*m*, 2H).

^{13}C NMR (75 MHz, $CDCl_3$) *δ* (ppm) 141.7, 141.6 131.7, 131.4, 130.9, 130.6, 129.6, 128.1, 127.8, 127.5, 127.4, 126.2, 125.5, 125.5, 125.1, 124.8, 124.6, 123.4, 99.9, 99.8, 72.8, 72.5, 68.3, 66.3.

HRMS (ESI$^+$): exact mass calculated for $[M+H]^+$ ($C_{24}H_{19}SO_3^+$) requires m/z 387.1055 found m/z 387.1055

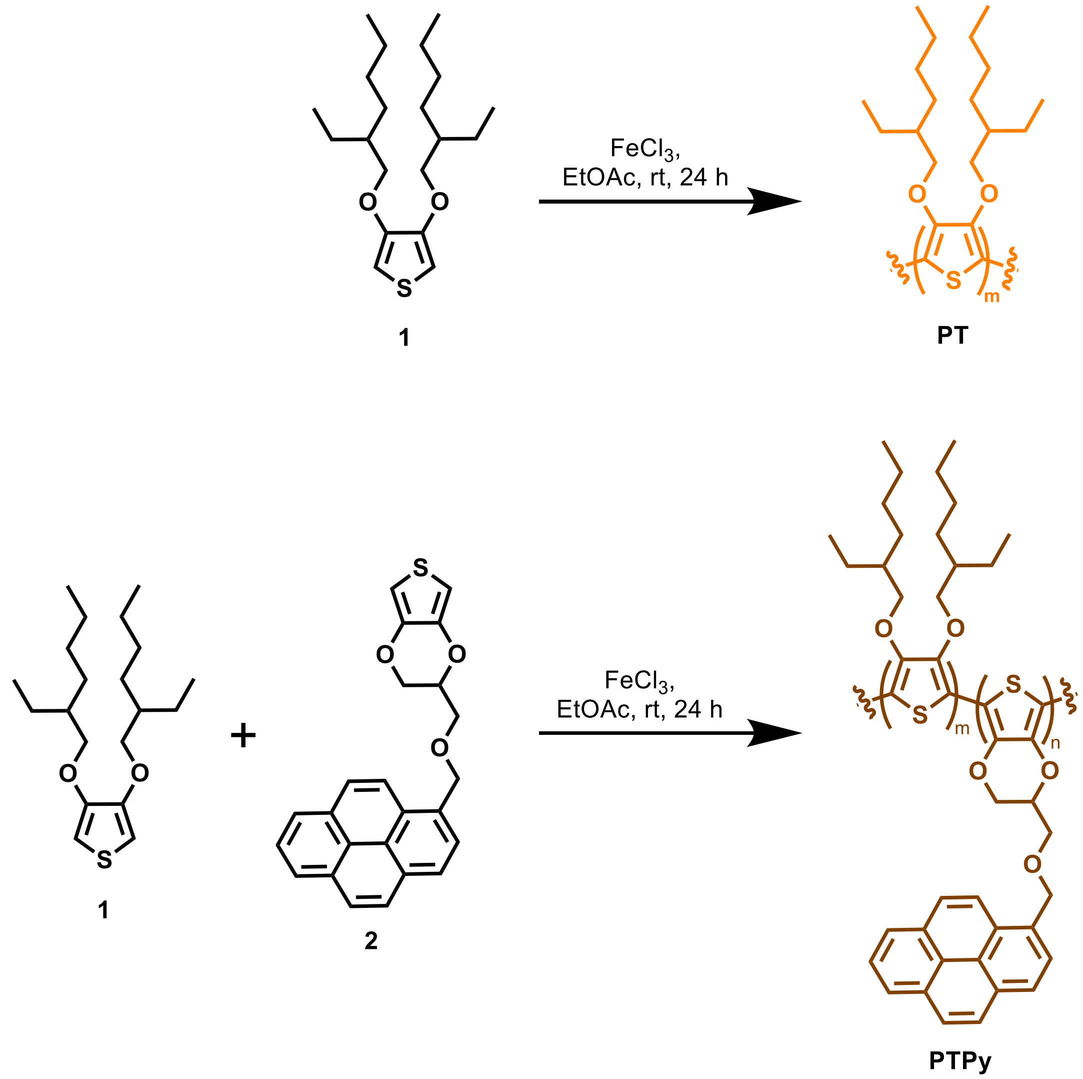

Scheme S2. Synthetic scheme for polymers **PT** and **PTPy**.

Synthesis of polymer PT. To a solution of **1** (100 mg, 0.29 mmol) in ethyl acetate (5 mL), a solution of iron chloride (147 mg, 0.9 mmol) in ethyl acetate (5 mL) was slowly added. The reaction mixture was stirred for 24 h at room temperature. MeOH (50 mL) was then added to the mixture and the solution was filtered. The solid was dissolved in chloroform (10 mL) and hydrazine (1 mL) was slowly added to the mixture which was stirred for an additional 10 min. MeOH (50 mL) was added and the solution was filtered. The solid was dissolved again in chloroform (15 mL), MeOH (100 mL) was added and the solution was filtered. The solid was extracted into a small flask and dried to give the desired polymer as a reddish viscous solid (31.1 mg, 31.1 %).

^{1}H NMR (700 MHz, $CDCl_3$, 50 °C) δ (ppm) 3.95 (*br*, Th-OCH_2-), 1.78 (*br*, -CH-), 1.53-1.29 (*br*, -CH_2-), 0.91-0.86 (*br*, -CH_3). Data is consistent with the previously reported spectrum.[1]

Synthesis of copolymer *PTPy.* To a solution of **1** (88.1 mg, 0.259 mmol) and **2** (10 mg, 0.0259 mmol) in ethyl acetate (2.5 mL) a solution of iron chloride (138.5 mg, 0.854 mmol) in ethyl acetate (2.5 mL) was slowly added. The reaction mixture was stirred for 24 h at room temperature. MeOH (50 mL) was then added to the mixture and the solution was filtered. The solid was dissolved in chloroform (10 mL) and hydrazine (0.5 mL) was added slowly to the mixture which was stirred for an additional 10 min. MeOH (100 mL) was added and the solution was filtered. The solid was dissolved again in chloroform (10 mL), MeOH (100 mL) was added and the solution was filtered. The solid was dried to give the desired polymer as a reddish solid (17 mg, 18 %).

^{1}H NMR (700 MHz, $CDCl_3$, 50 °C) δ (ppm) 8.30-8.20 (*br*, Ar-H), 4.74-3.50 (*br*, -OCH_2), 1.71-1.58 (*br*, -CH-), 1.50-1.44 (*br*, -CH_2-), 1.35-1.28 (*br*, -CH_2-), 0.92-0.84 (*br*, -CH_3).

2. NMR spectra

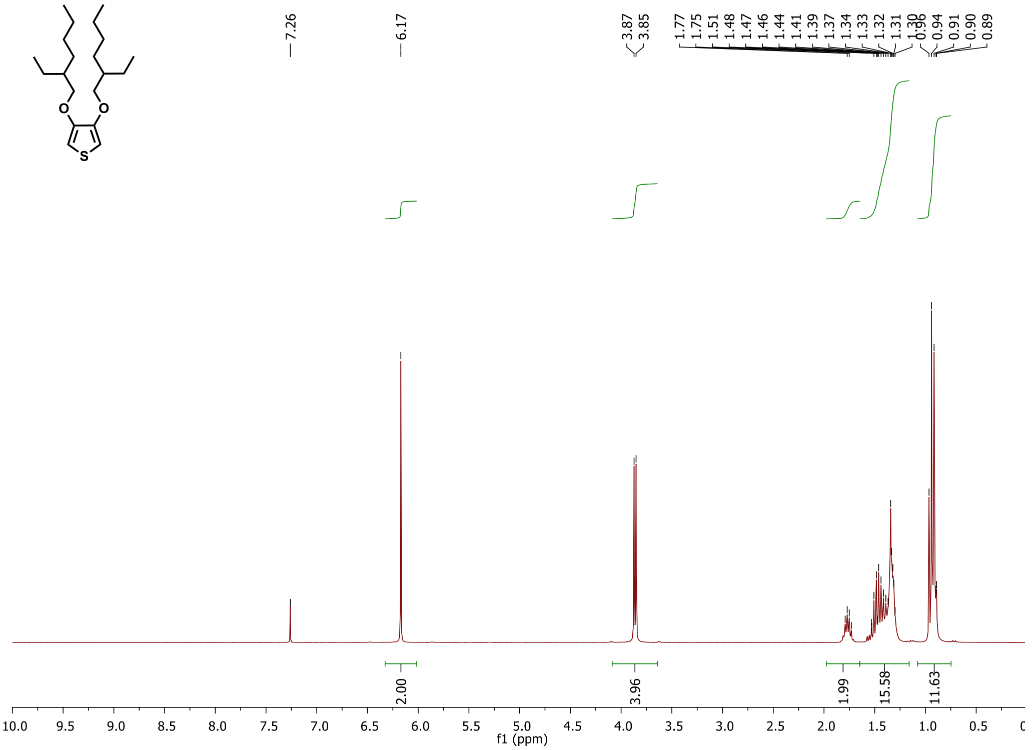

Figure S1. ^{1}H NMR spectrum of **1** in $CDCl_3$.

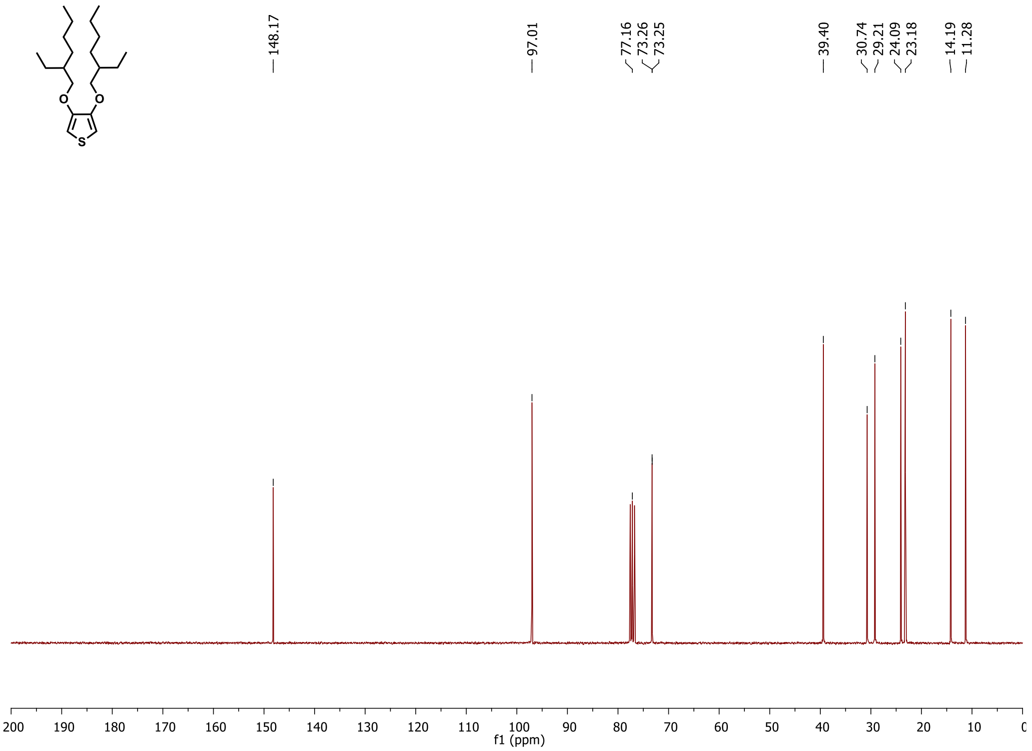

Figure S2. ^{13}C NMR spectrum of **1** in $CDCl_3$.

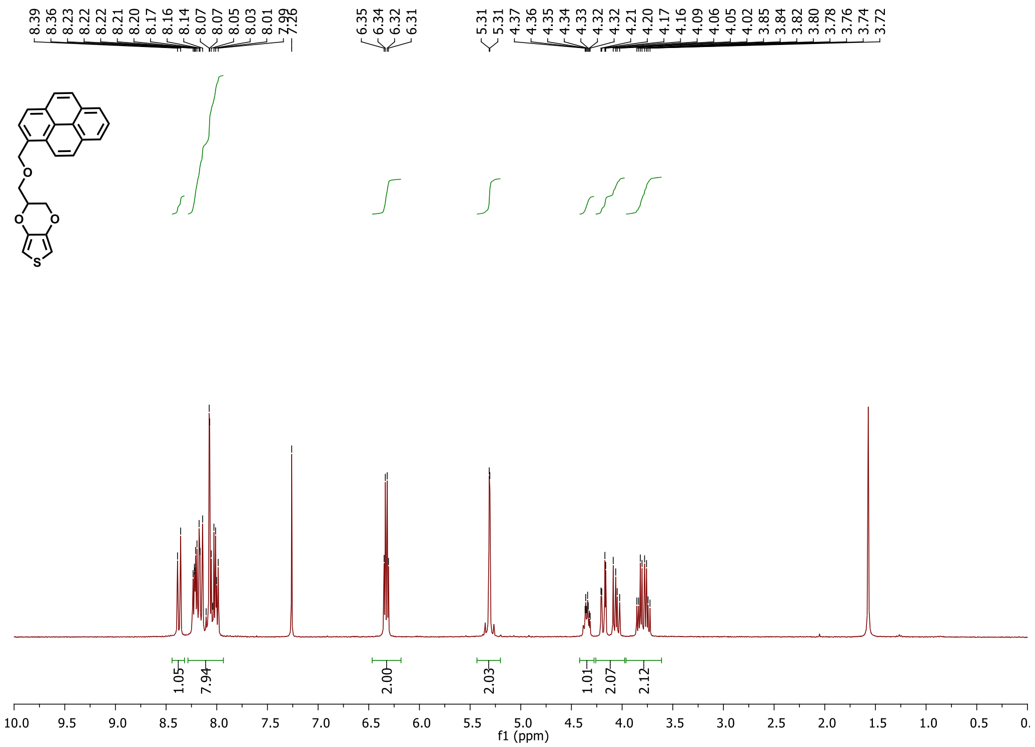

Figure S3. ^{1}H NMR spectrum of **2** in $CDCl_3$.

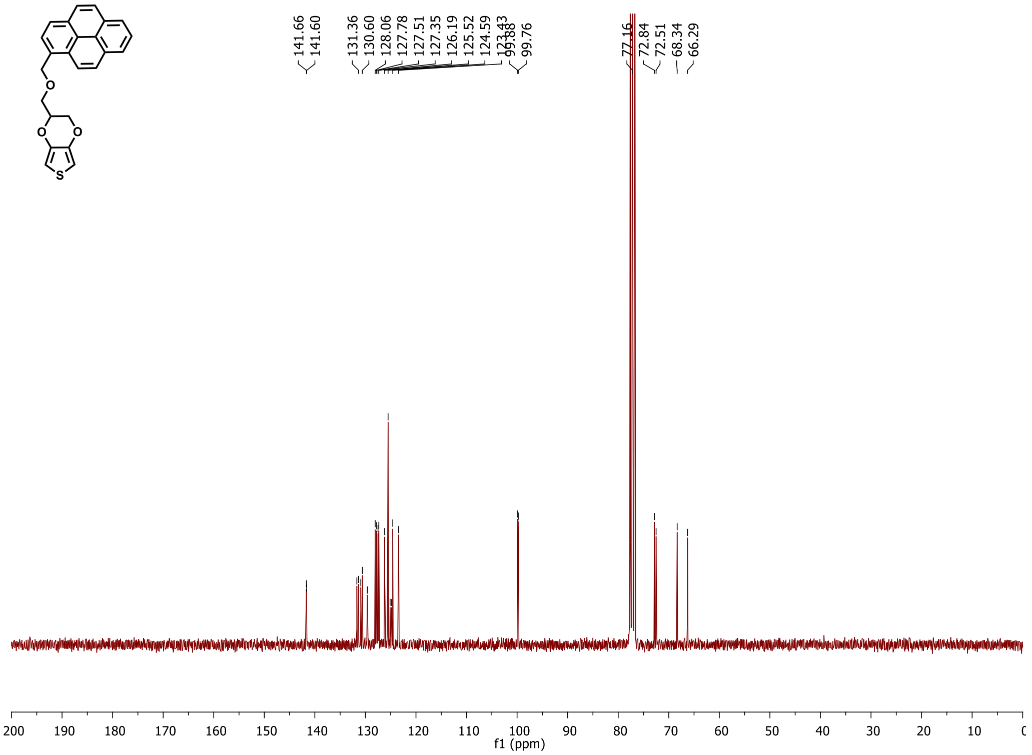

Figure S4. ^{13}C NMR spectrum of **2** in $CDCl_3$.

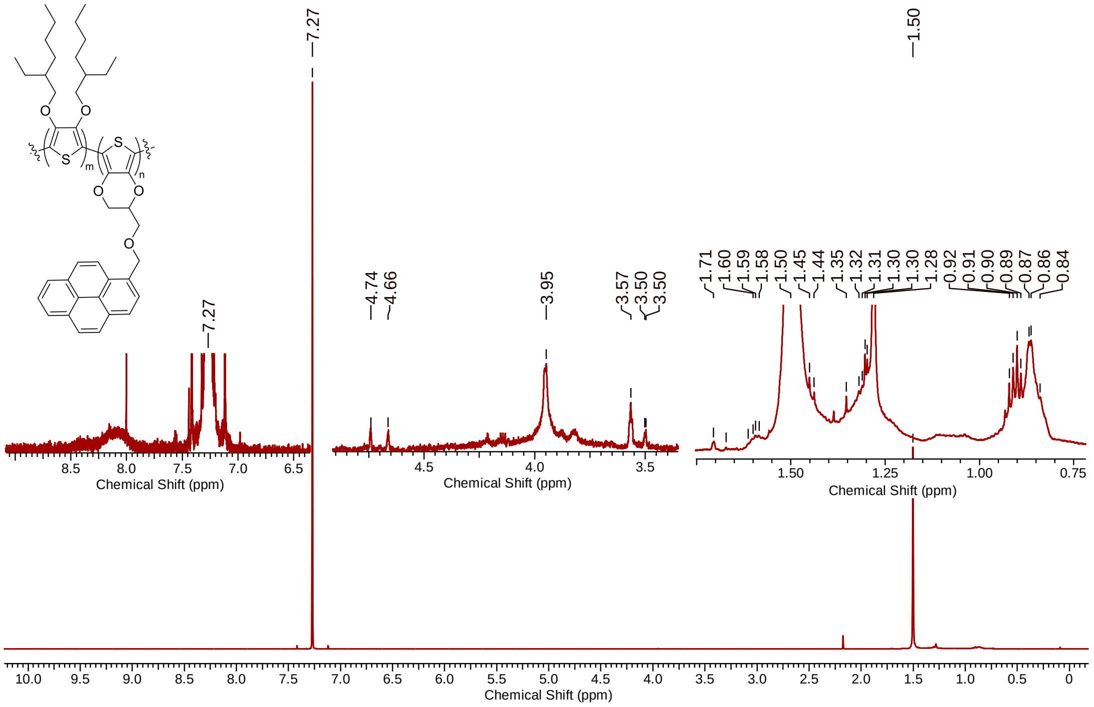

Figure S5. ^{1}H NMR spectrum of **PTPy** in $CDCl_3$ (700 MHz at 50 °C).

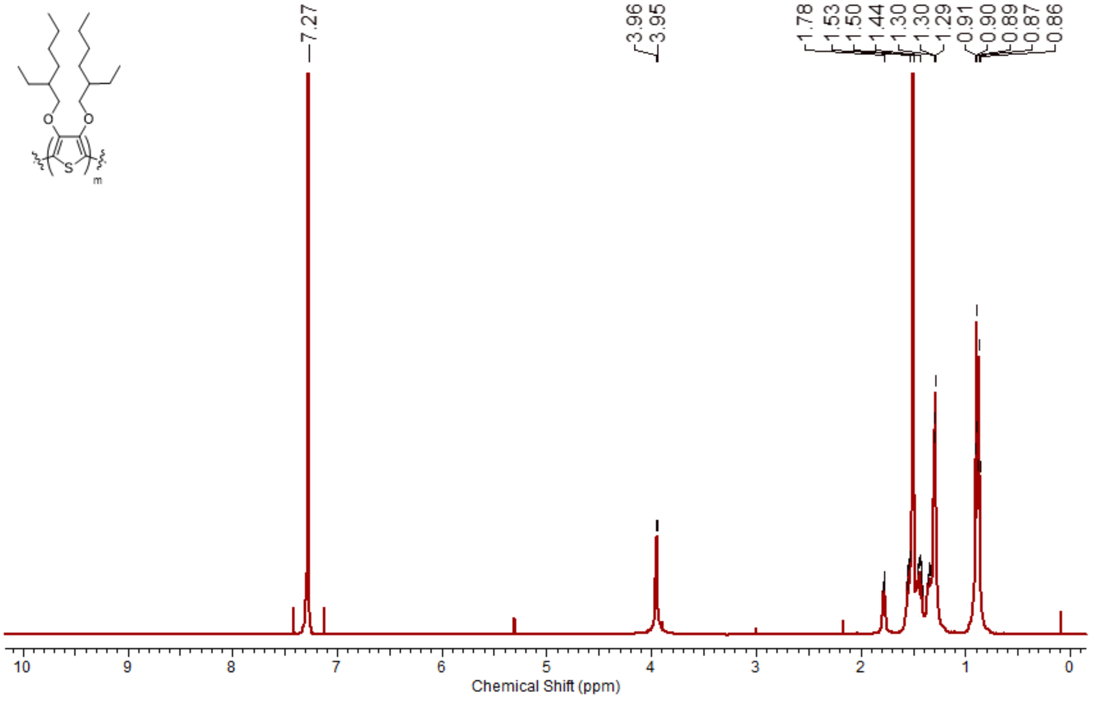

Figure S6. ^{1}H NMR spectrum of **PT** in $CDCl_3$ (700 MHz at 50 °C).

3. Supporting figures and tables

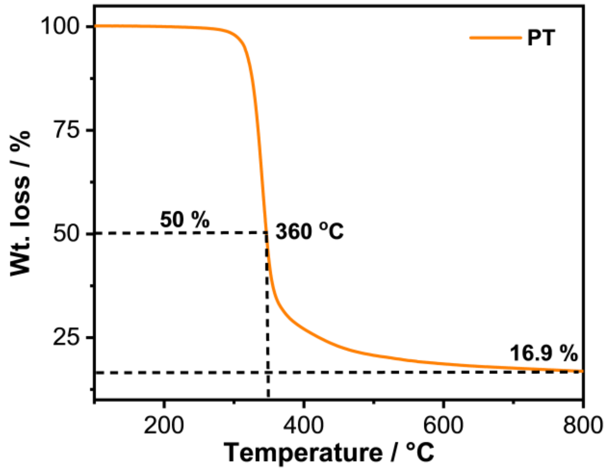

Figure S7. TGA profile of **PT** recorded at 10 °C/min under N_2 atmosphere.

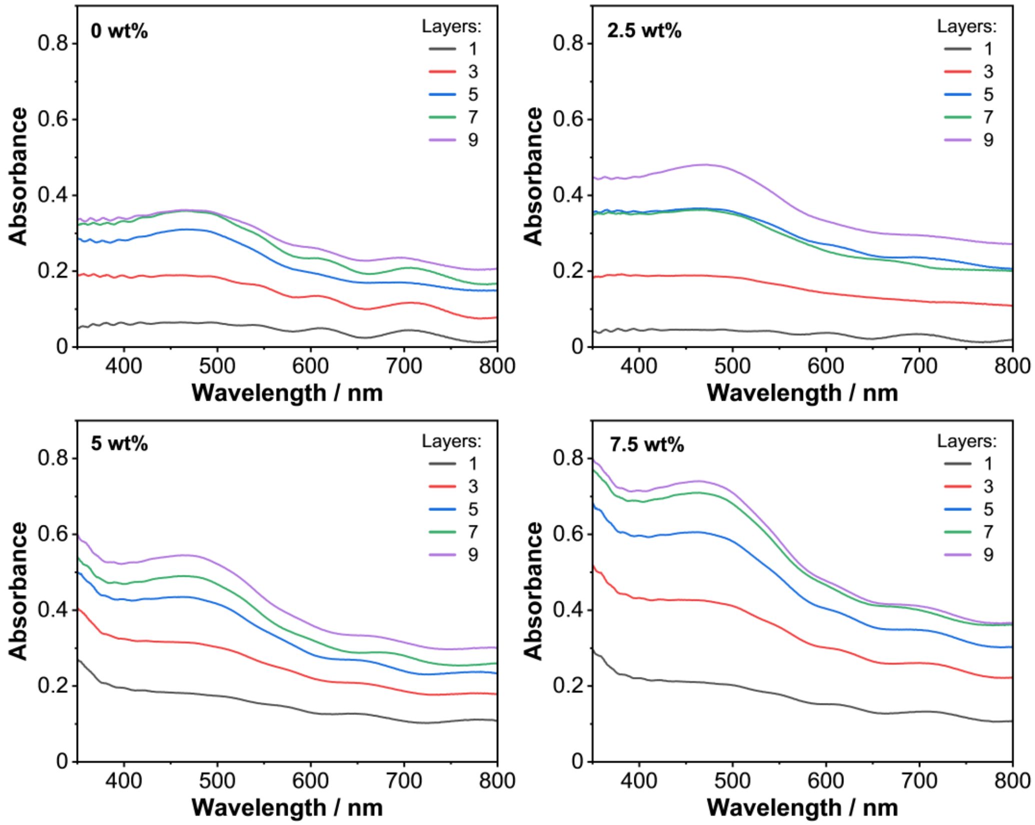

Figure S8. UV–VIS absorption spectra of **PTPy/MWCNTs** (0–7.5 wt%) films spray coated onto PET substrates, recorded as a function of the number of layers.

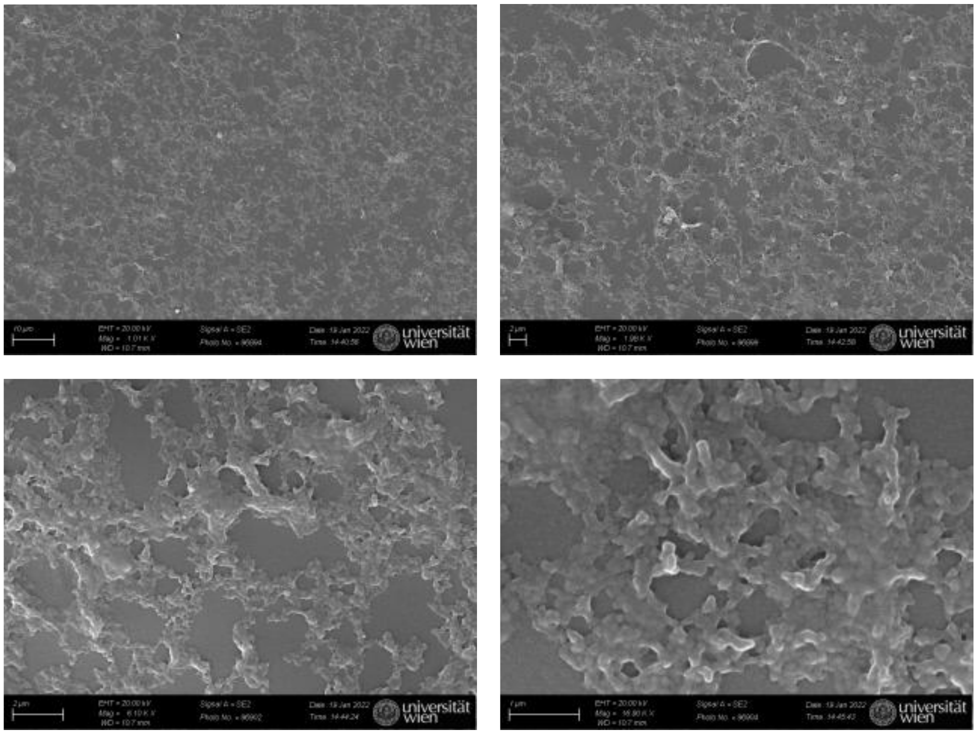

Figure S9. SEM images taken with different magnifications of a **PTPy** film spray-coated onto a Si support.

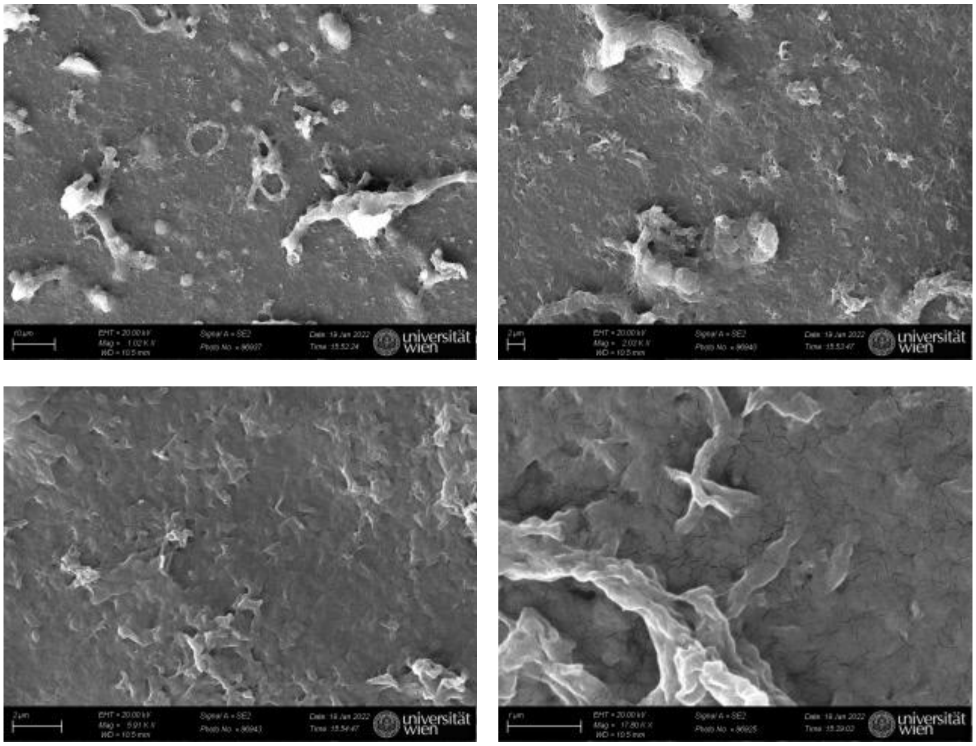

Figure S10. SEM images taken with different magnifications of a **PTPy/MWCNTs** (7.5 wt%) film spray-coated onto a Si support.

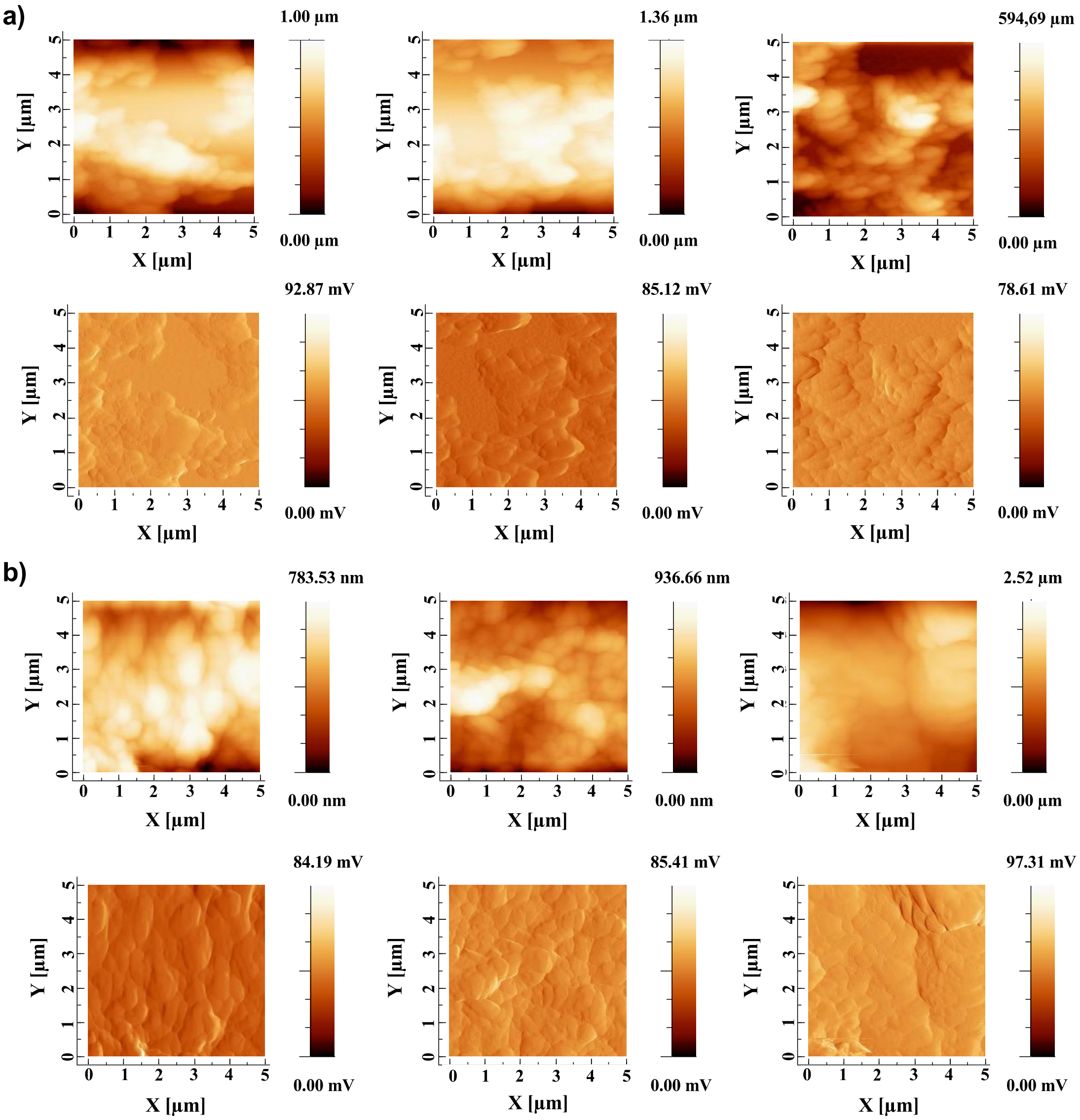

Figure S11. AFM topography (top) and amplitude (bottom) images of a) **PTPy** and b) **PTPy/MWCNTs** 7.5 wt% films spray-coated onto a PET-ITO electrode.

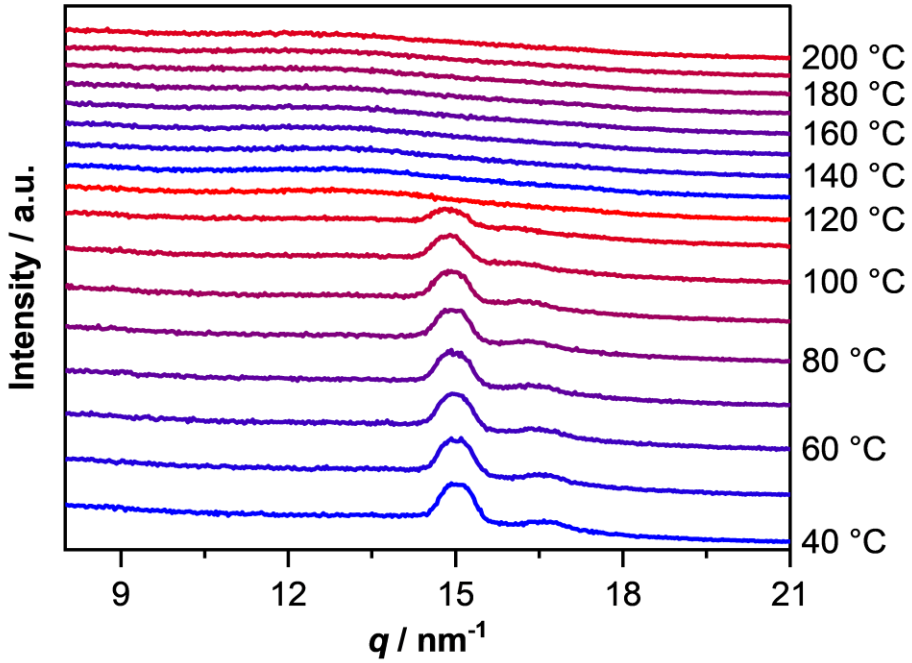

Figure S12. GIWAXS patterns of **PTPy/MWCNTs** (7.5 wt%) blend spray-coated on silicon support measured during heating ramp from 40 to 200 ^{o}C (10 ^{o}C min^{-1}).

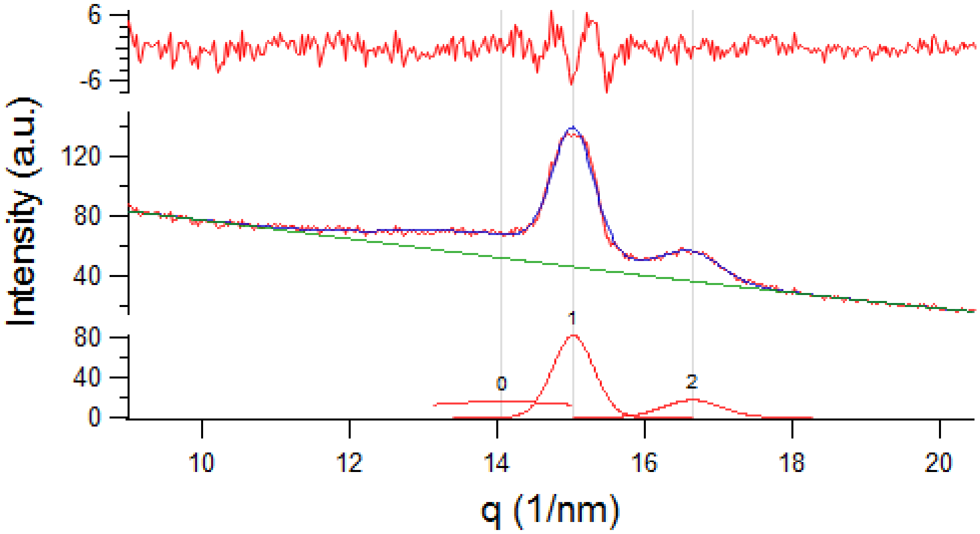

Figure S13. GIWAXS patterns of **PTPy/MWCNTs** (7.5 wt%) blend spray-coated on silicon support at rt. Top panel: residuals between the fit (red) and the data, middle panel: fit of the three Gaussian contributions for the backbone order (peak 0), hydrocarbon chain packing (peak 1), and π–π stacking interactions (peak 2) data (red), fit (blue), 3rd order polynomial background (green). The lower panel displays the single contributions of the Gaussian peaks of the GIWAXS profile.

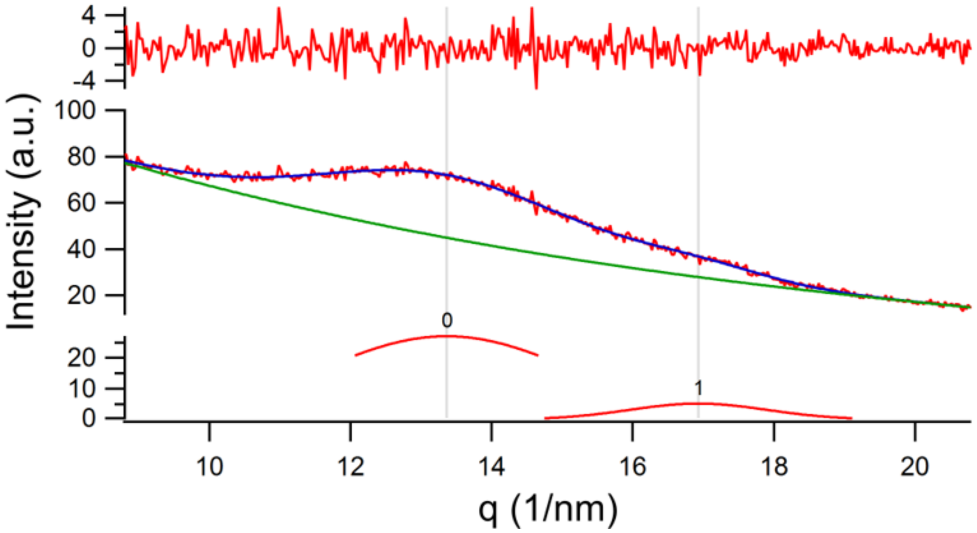

Figure S14. GIWAXS patterns of **PTPy/MWCNTs** (7.5 wt%) blend spray-coated on silicon support at 120 °C. Top panel: residuals between the fit (red) and the data, middle panel: fit of the three Gaussian contributions for the backbone order (peak 0), hydrocarbon chain packing/ π–π stacking interactions (peak 1) data (red), fit (blue), 3rd order polynomial background (green). The lower panel displays the single contributions of the Gaussian peaks of the GIWAXS profile.

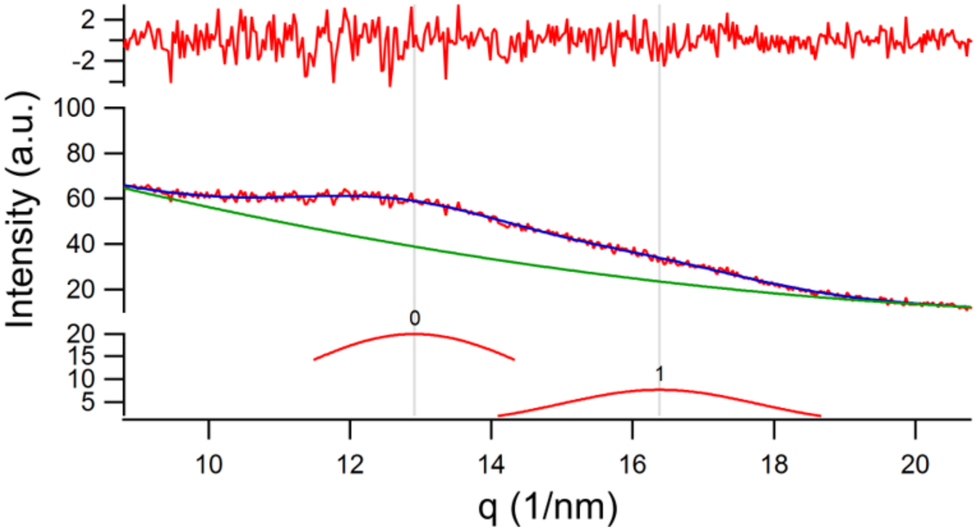

Figure S15. GIWAXS patterns of **PTPy/MWCNTs** (7.5 wt%) blend spray-coated on silicon support at 200 °C. Top panel: residuals between the fit (red) and the data, middle panel: fit of the three Gaussian contributions for the backbone order (peak 0), hydrocarbon chain packing/π–π stacking interactions (peak 1) data (red), fit (blue), 3rd order polynomial background (green). The lower panel displays the single contributions of the Gaussian peaks of the GIWAXS profile.

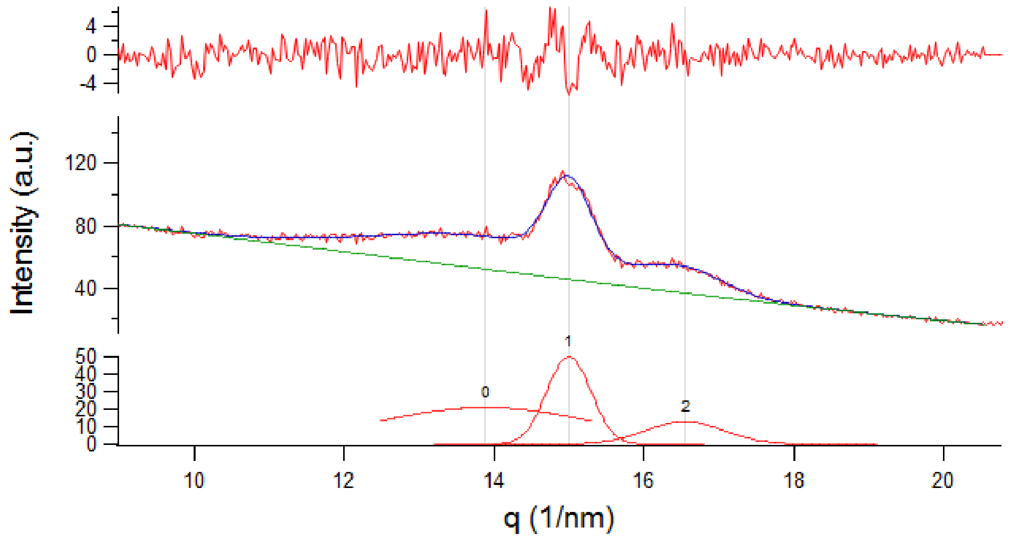

Figure S16. GIWAXS patterns of **PTPy/MWCNTs** (7.5 wt%) blend spray-coated on silicon support at rt after annealing. Top panel: residuals between the fit (red) and the data, middle panel: fit of the three Gaussian contributions for the backbone order (peak 0), hydrocarbon chain packing (peak 1), and π–π stacking interactions (peak 3) data (red), fit (blue), 3rd order polynomial background (green). The lower panel displays the single contributions of the Gaussian peaks of the GIWAXS profile.

Table S1. Results of the multipeak fitting of **PTPy/MWCNTs** at various temperatures and after annealing.

			**Location q (1/nm)**	**Loc Sigma**	**Amplitude**	**Amp Sigma**	**Area**	**Area Sigma**	**FWHM**	**FWHM Sigma**	**d-spacing (nm)**
G7_1M_cS1_ic	Peak0	Gauss	14.07	0.08	15.68	0.90	55.17	6.58	3.31	0.230	0.447
RT	Peak1	Gauss	15.04	0.00	81.23	0.92	57.16	1.22	0.66	0.009	0.418
	Peak2	Gauss	16.66	0.02	17.18	0.77	16.92	1.45	0.93	0.051	0.377
G8_1M_00008_cS1_ic	Peak0	Gauss	13.36	0.04	27.03	2.36	121.47	16.76	4.22	0.228	0.470
120 °C	Peak1	Gauss	16.93	0.10	5.17	0.69	12.64	2.95	2.30	0.268	0.371
G8_1M_00008_cS1_ic	Peak0	Gauss	12.91	0.10	19.76	4.41	86.30	31.01	4.10	0.580	0.487
200 °C	Peak1	Gauss	16.38	0.28	7.69	1.66	26.81	9.50	3.28	0.484	0.384
G16_1M_cS1_ic	Peak0	Gauss	13.89	0.08	21.11	1.23	78.00	10.27	3.47	0.271	0.452
annealed	Peak1	Gauss	15.00	0.00	50.33	1.19	36.93	1.50	0.69	0.015	0.419
	Peak2	Gauss	16.55	0.03	13.07	1.16	16.91	2.46	1.22	0.084	0.380

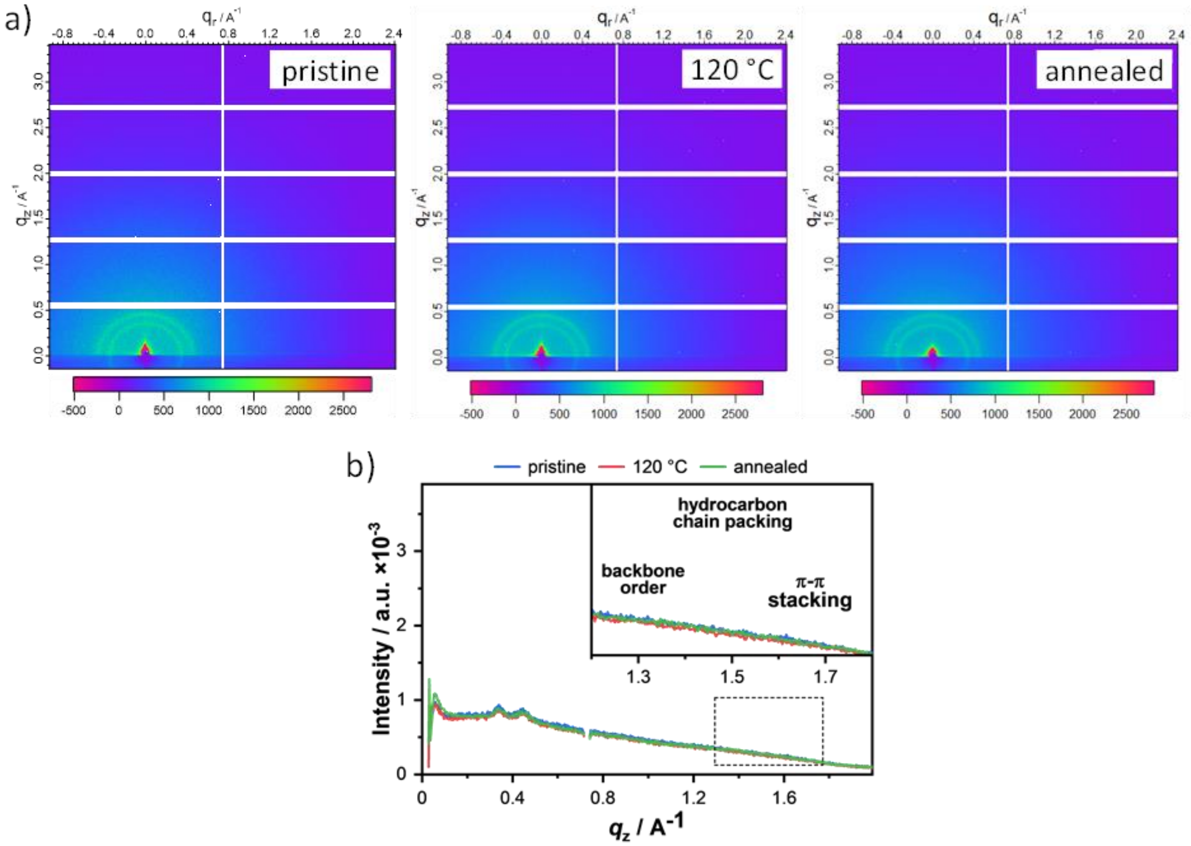

Figure S17. a) 2D-GIWAXS images of **PT/MWCNT** (7.5 wt%) spray-coated on a silicon chip at rt, upon heating up to 120 °C (2 °C/min), and subsequent cooling back to rt. b) Radially integrated intensity profile of the corresponding GIWAXS images.

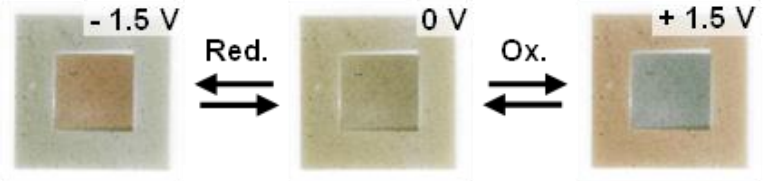

Figure S18. Square pattern of the 1 cm^2 ECD built with **PTPy/MWCNTs** (7.5 wt%) used for characterization of the electrochromic performance.

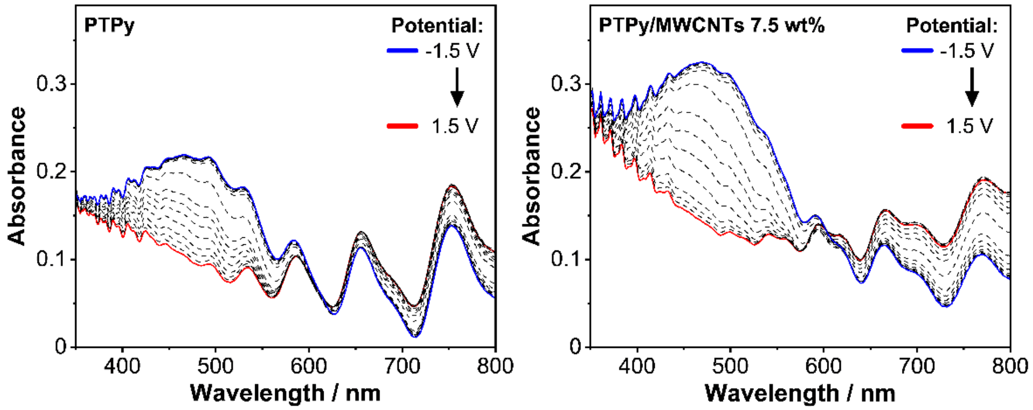

Figure S19. Spectroelectrochemistry recorded for devices built with **PTPy** (left) and **PTPy/MWCNTs** (7.5 wt%, right).

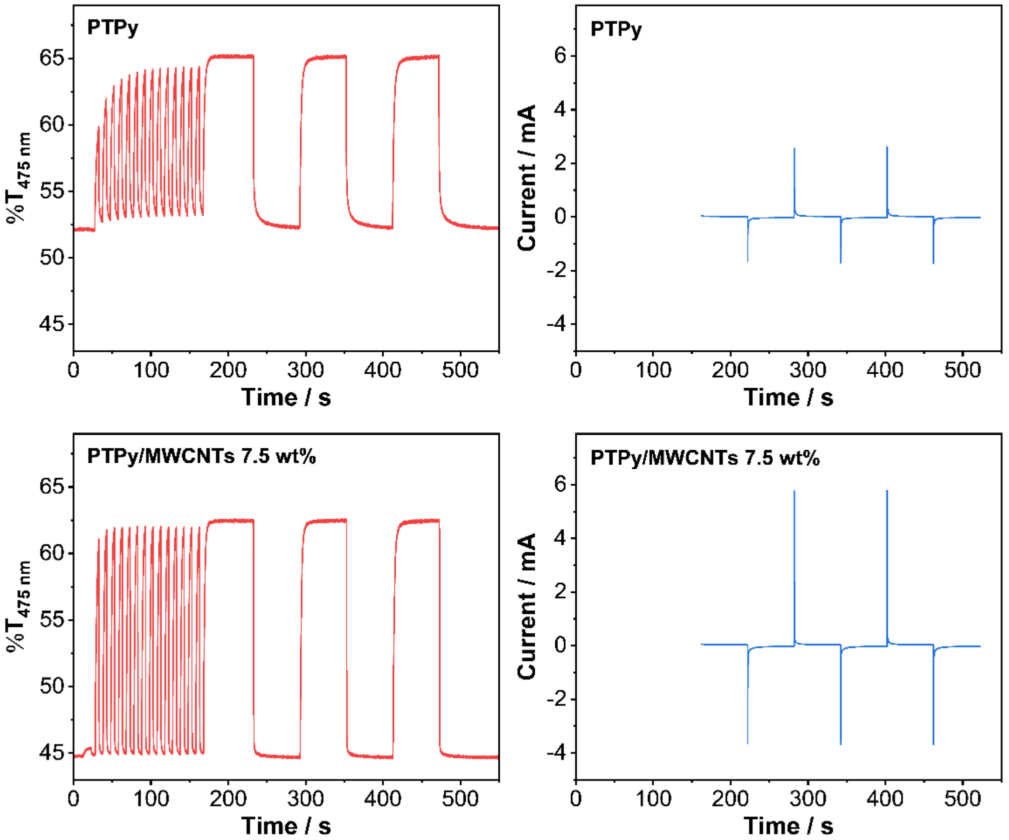

Figure S20. Transmittance change (left) and chronoamperometry (right) during electrochemical switching of devices assembled with **PTPy** (top) and **PTPy/MWCNTs** (7.5 wt%, bottom).

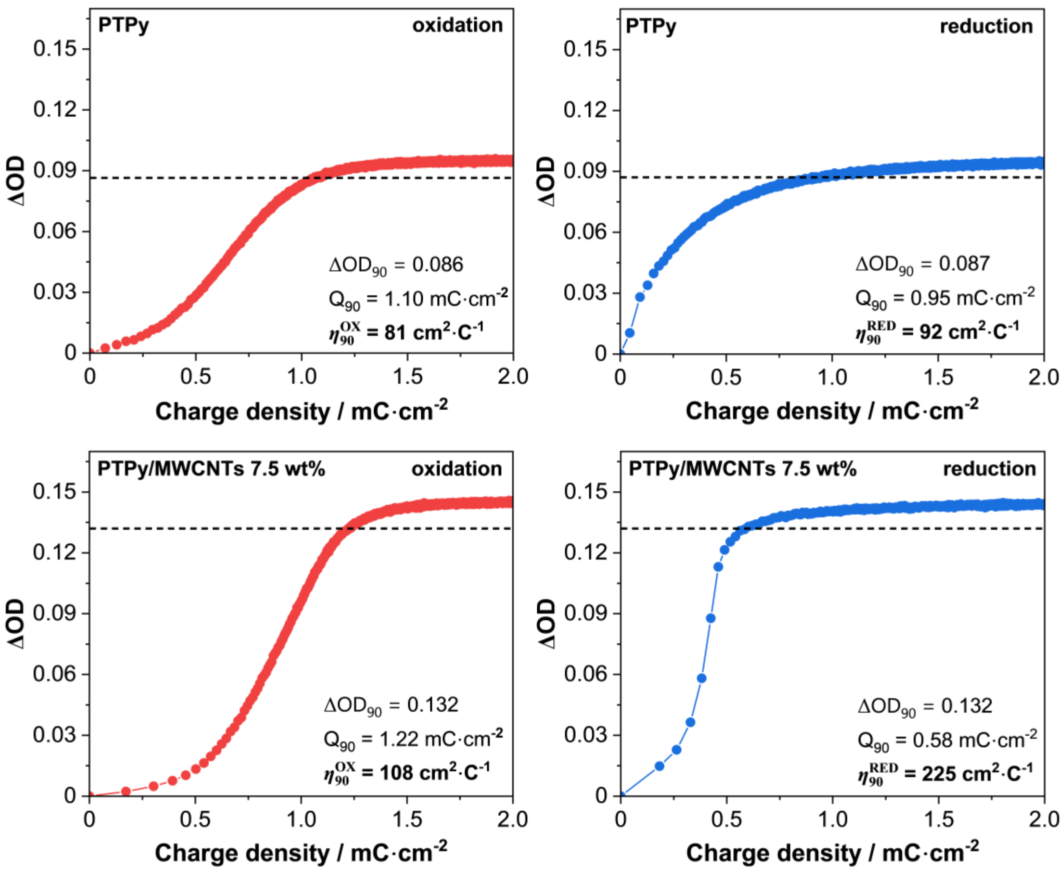

Figure S21. Change in optical density as a function of the charge density consumed during electrochemical oxidation (left) and reduction (right) of devices assembled with **PTPy** (top) and **PTPy/MWCNTs** (7.5 wt%, bottom).

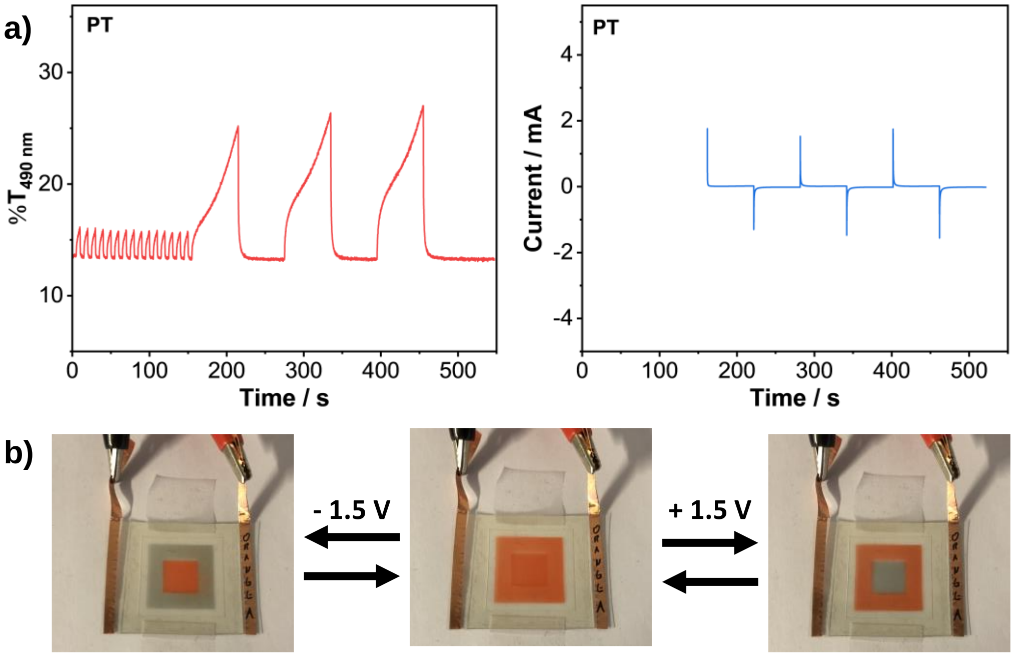

Figure S22. a) Transmittance change (left) and chronoamperometry (right) during electrochemical switching of a device assembled with **PT**, and b) day-light pictures in its different states.

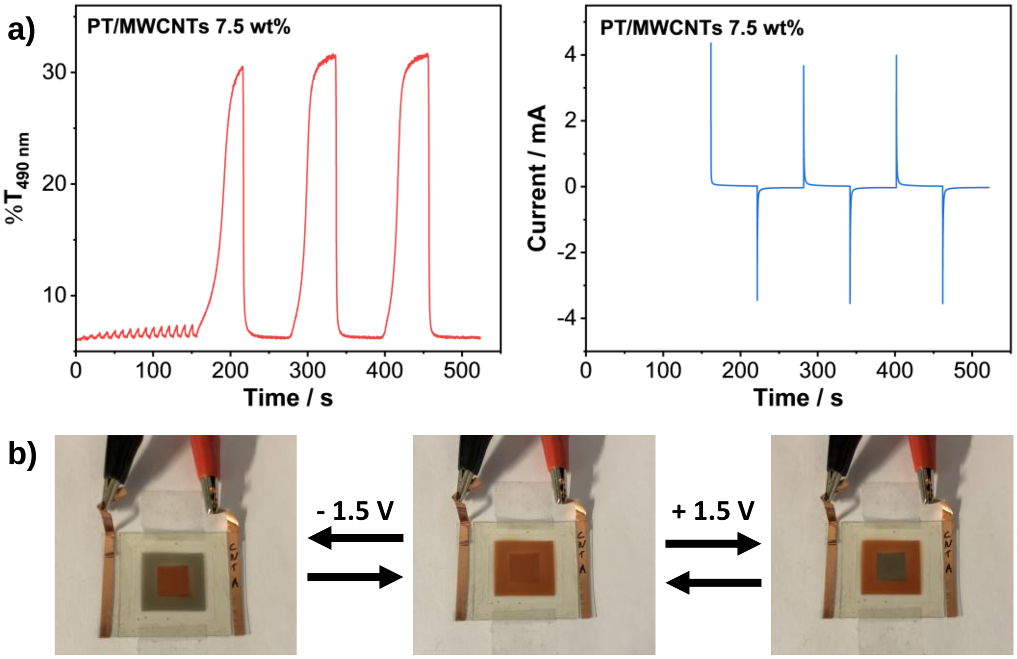

Figure S23. a) Transmittance change (left) and chronoamperometry (right) during electrochemical switching of a device assembled with **PT/MWCNTs** (7.5 wt%), and b) day-light pictures in its different states.

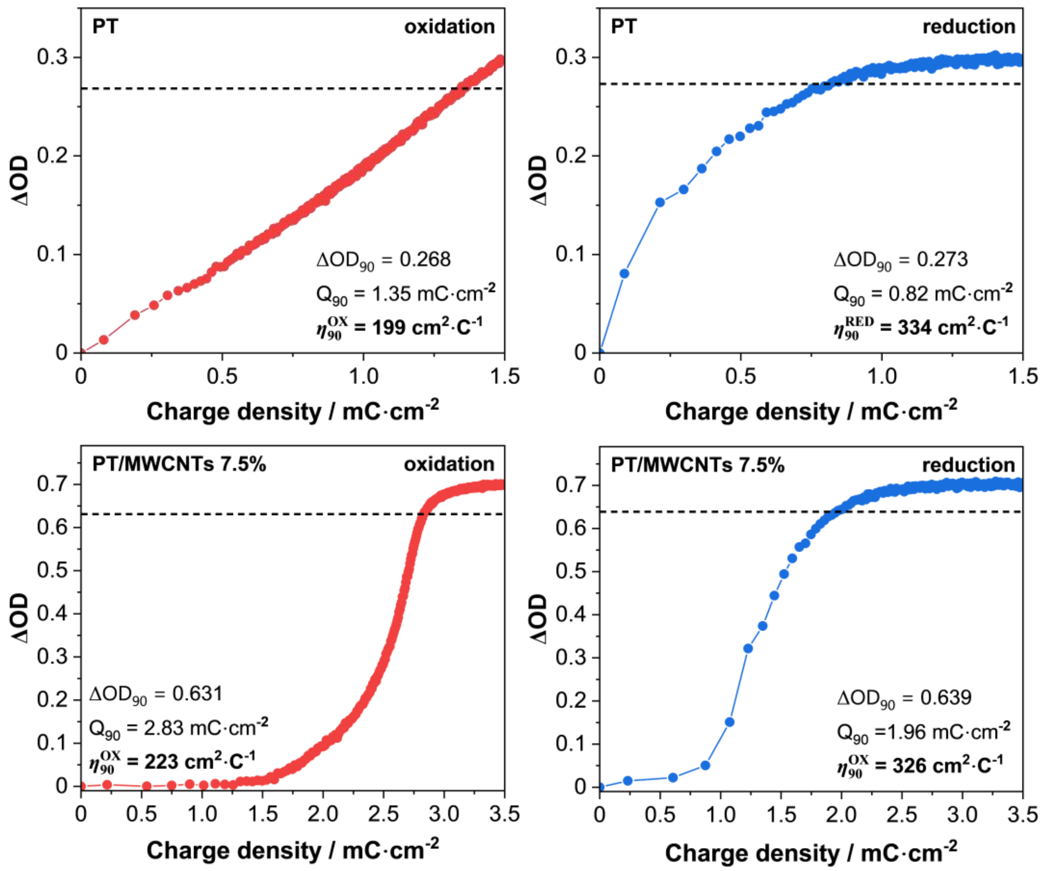

Figure S24. Change in optical density as a function of the charge density consumed during electrochemical oxidation (left) and reduction (right) of devices assembled with **PT** (top) and **PT/MWCNTs** (7.5 wt%, bottom).

Table S2. Color contrast, switching time, and coloration efficiency, for ECDs assembled with **PT** and **PT/MWCNTs**.

	**Δ%T**	t_{90}^{OX} **(s)**	t_{90}^{RED} **(s)**	η_{90}^{OX} **(cm^2 C^{-1})**	η_{90}^{RED} **(cm^2 C^{-1})**
**PT**	13.4	53.2	2.5	199	334
**PT/MWCNT** (7.5 wt%)	25.4	25.8	1.7	223	326

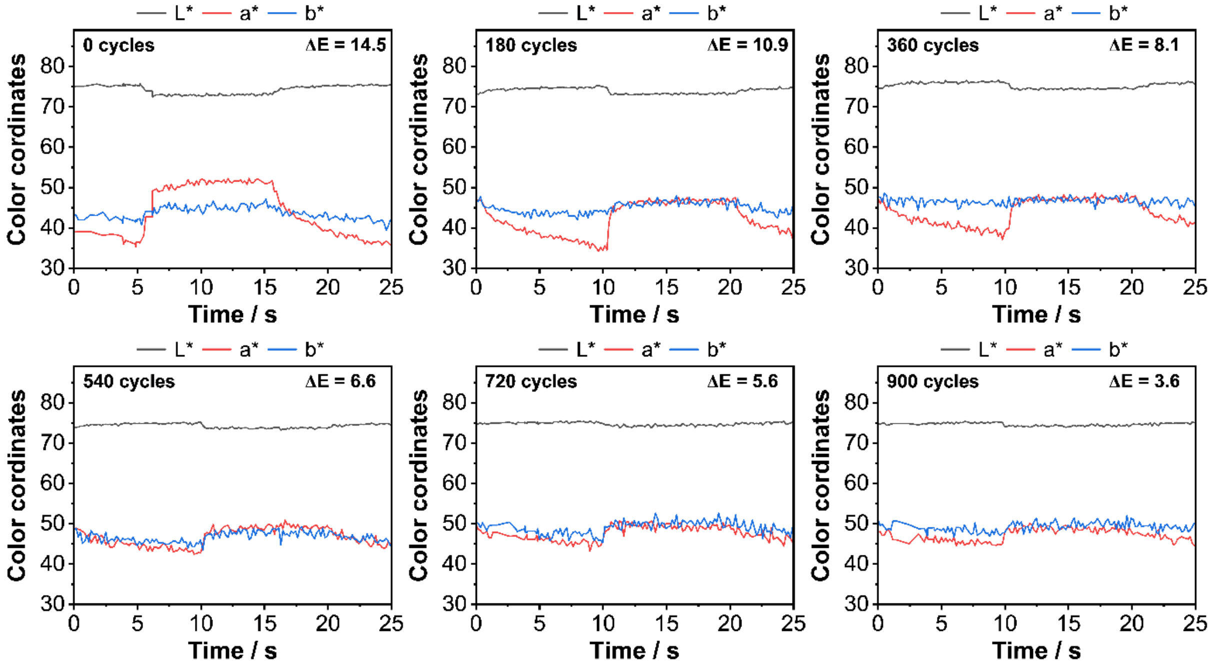

Figure S25. L*a*b* color coordinates monitored as a function of the number of electrochemical cycles (±1.5 V, 10 s) for a device assembled with **PT**.

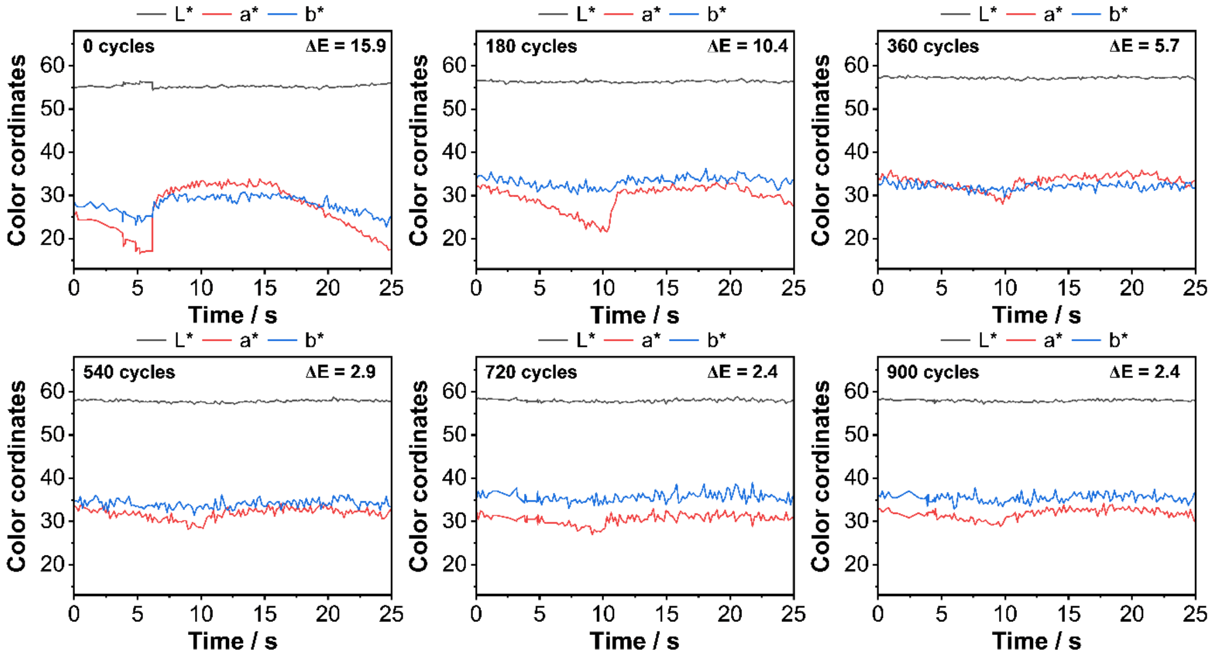

Figure S26. L*a*b* color coordinates monitored as a function of the number of electrochemical cycles (±1.5 V, 10 s) for a device assembled with **PT/MWCNTs** (7.5 wt%).

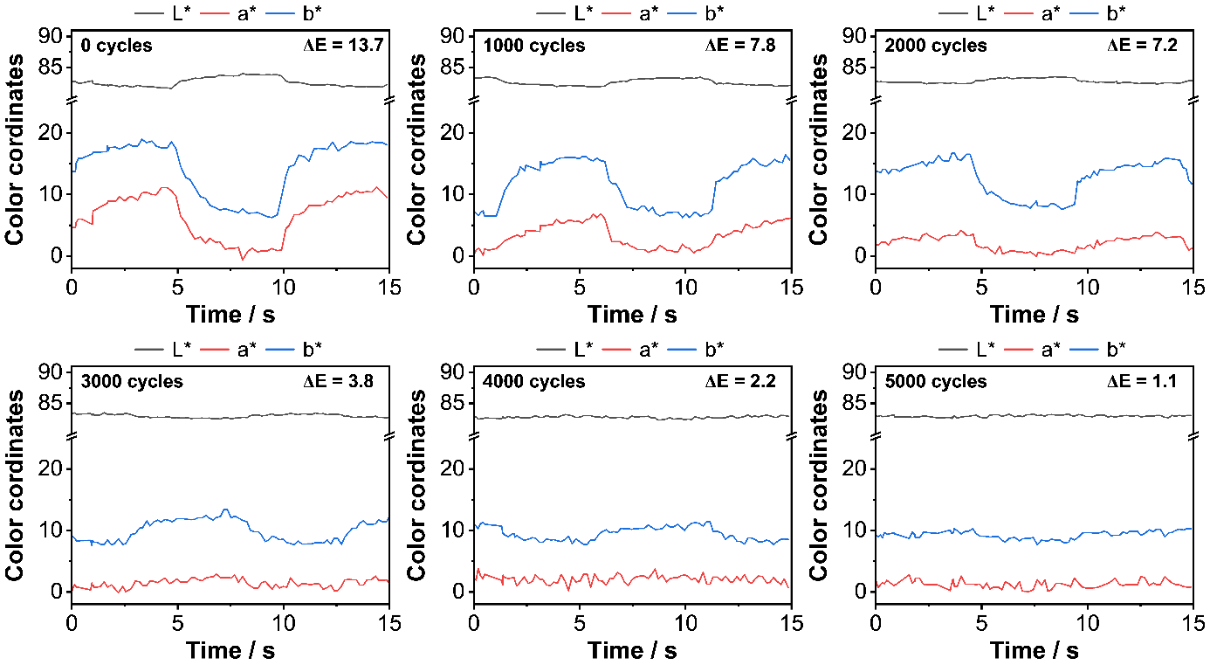

Figure S27. L*a*b* color coordinates monitored as a function of the number of electrochemical cycles (±1.5 V, 5 s) for a device assembled with **PTPy**.

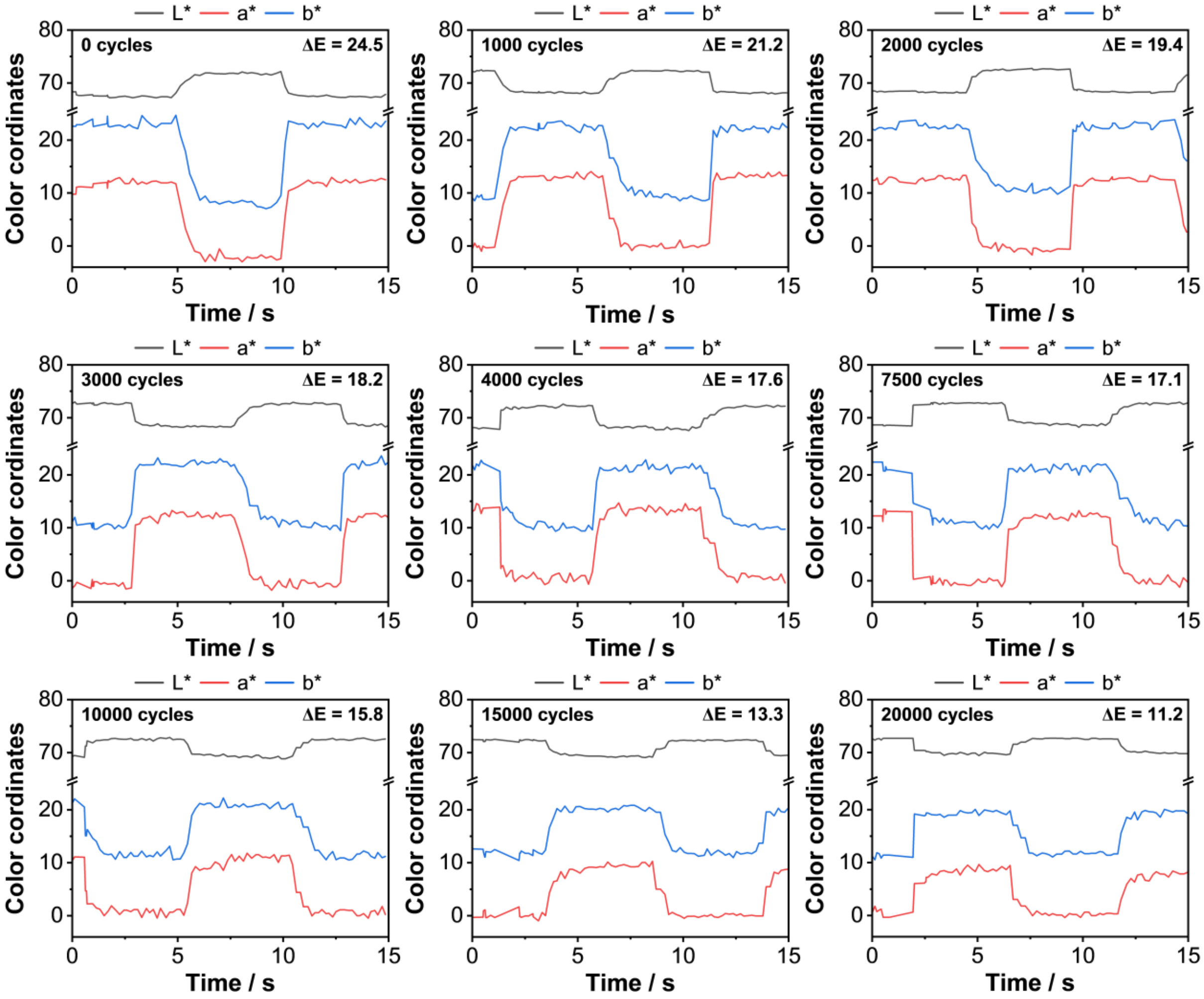

Figure S28. L*a*b* color coordinates monitored as a function of the number of electrochemical cycles (±1.5 V, 5 s) for a device assembled with **PTPy/MWCNTs** (7.5 wt%).

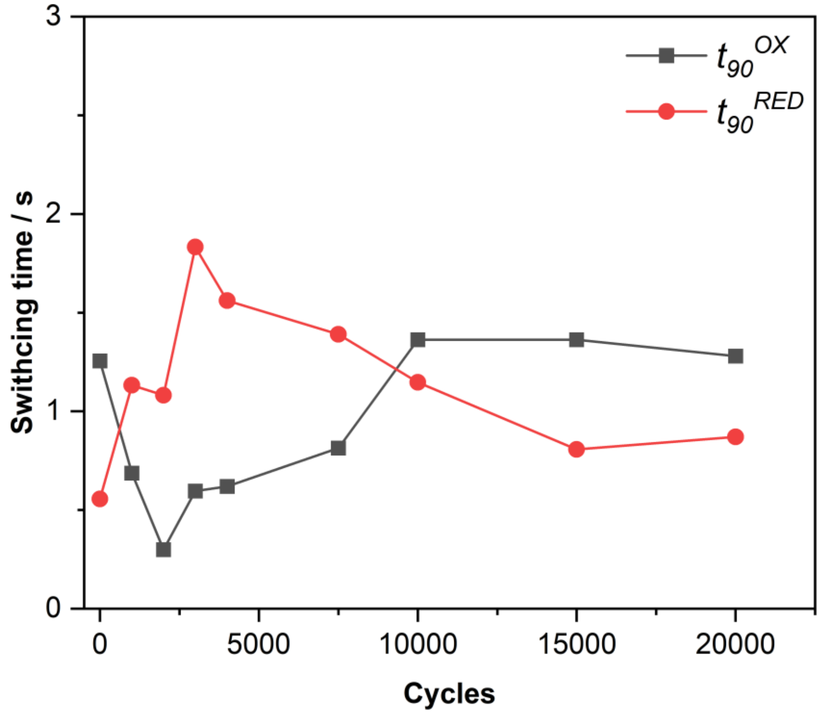

Figure S29. Oxidation and reduction **s**witching times monitored as a function of the number of electrochemical cycles (±1.5 V, 5 s) for a device assembled with **PTPy/MWCNTs** (7.5 wt%).

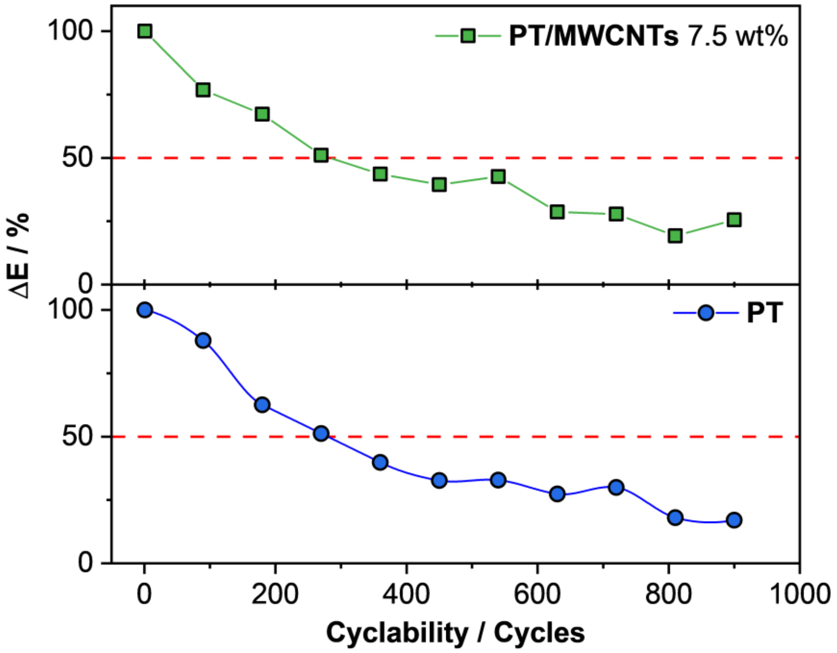

Figure S30. Cycling stability of **PT** and **PT/MWCNTs** (7.5 wt%) ECDs.

Table S3. Electrochromic properties of state-of-the-art flexible ECDs based on polythiophenes and other organic compounds and their hybrids.

Reference	EC material	Δ%T	t_{90}^{OX} / s	t_{90}^{RED}/ s	Stability / nº cycles
**This work**	**PTPy/MWCNTs**	**17.8 (475 nm)**	**3.6**	**0.3**	**369 (95 % ΔE)<br>737 (90 % ΔE)<br>2810 (75 % ΔE)<br>17600 (50 % ΔE)**
Ref[2]	Viologen-MoS_2-CNTs/Polythiophene	12.7 (515 nm)	0.47	0.8	200 (≈92% Δ%T)
Ref[3]	PSF P3HT	≈ 64.6 (520 nm)	125	22	500 (≈ 94% Δ%T)
Ref[4]	PEDOT/Prussian blue	≈ 43.0 (635 nm)	≈ 10	≈ 8	100 (97% ΔI)
Ref[5]	PEDOT: PSS Ni grid	40 (650 nm)	4.7	2.7	1000 (80% Δ%T)
Ref[6]	Viologen Ag grid	46.7 (615 nm)	5	12	200 (86% Δ%T)
Ref[7]	Fluorinated Polythiophene	49 (530 nm)	1.9	2.2	1000 (95% Δ%T)
Ref[8]	Polythiophene/ PCBM	13 (550 nm)	≈2.8	1	250 (≈ 96% Δ%T)

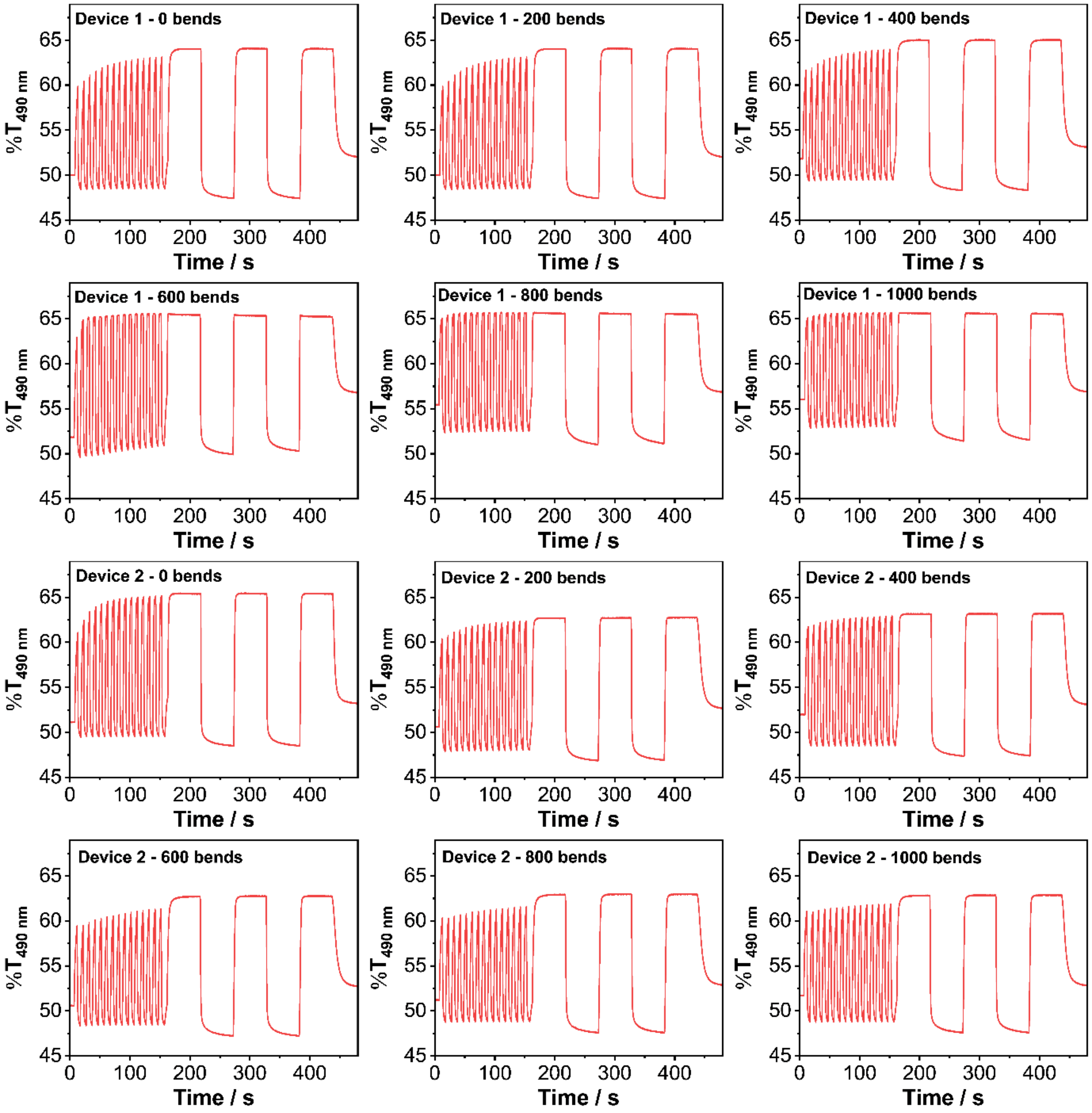

Figure S31. Transmittance change during electrochemical switching for two ECDs assembled with **PTPy/MWCNTs** (7.5 wt%), as a function of the number of bends (bend radius = 10 mm).

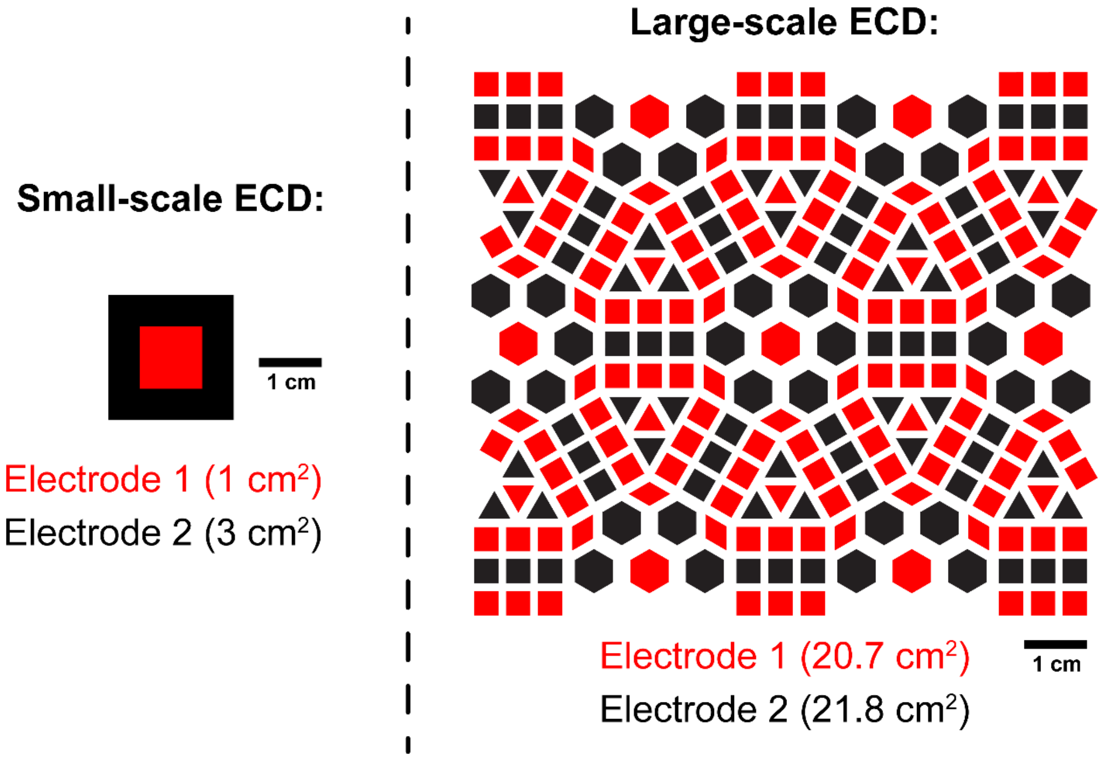

Figure S32. Patterns used to manufacture small-scale (left) and large-scale (right) ECDs.

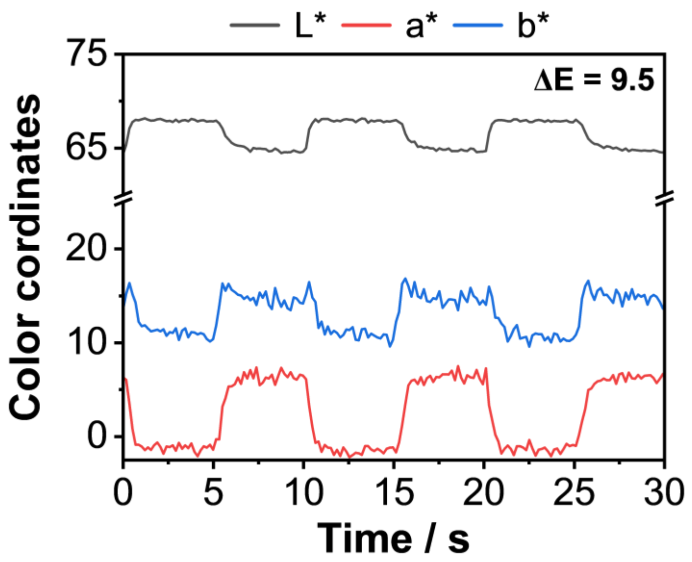

Figure S33. L*a*b* color coordinates monitored during electrochemical switching (±1.5 V, 5 s) for a large-area (11×13 cm) device assembled with **PTPy/MWCNTs** (7.5 wt%).

4. References

(1) Dyer, A. L.; Craig, M. R.; Babiarz, J. E.; Kiyak, K.; Reynolds, J. R. Orange and Red to Transmissive Electrochromic Polymers Based on Electron-Rich Dioxythiophenes. *Macromolecules* **2010**, *43* (10), 4460-4467. DOI: 10.1021/ma100366y.

(2) Kandpal, S.; Ghosh, T.; Rani, C.; Tanwar, M.; Sharma, M.; Rani, S.; Pathak, D. K.; Bhatia, R.; Sameera, I.; Jayabalan, J.; et al. Bifunctional Application of Viologen-MoS2-CNT/Polythiophene Device as Electrochromic Diode and Half-Wave Rectifier. *ACS Mater. Au* **2022**, *2* (3), 293-300. DOI: 10.1021/acsmaterialsau.1c00064.

(3) Chaudhary, A.; Sivakumar, G.; Pathak, D. K.; Tanwar, M.; Misra, R.; Kumar, R. Pentafluorophenyl substituted fulleropyrrolidine: a molecule enabling the most efficient flexible electrochromic device with fast switching. *J. Mater. Chem. C* **2021**, *9* (10), 3462-3469. DOI: 10.1039/d0tc04991c.

(4) Macher, S.; Schott, M.; Sassi, M.; Facchinetti, I.; Ruffo, R.; Patriarca, G.; Beverina, L.; Posset, U.; Giffin, G. A.; Löbmann, P. New Roll-to-Roll Processable PEDOT-Based Polymer with Colorless Bleached State for Flexible Electrochromic Devices. *Adv. Funct. Mater.* **2020**, *30* (6), 1906254. DOI: 10.1002/adfm.201906254.

(5) Zhao, S.-Q.; Liu, Y.-H.; Ming, Z.; Chen, C.; Xu, W.-W.; Chen, L.; Huang, W. Highly flexible electrochromic devices enabled by electroplated nickel grid electrodes and multifunctional hydrogels. *Optics Express* **2019**, *27* (21), 29547-29557. DOI: 10.1364/OE.27.029547.

(6) Lee, J.; Lee, Y.; Ahn, J.; Kim, J.; Yoon, S.; Kim, Y. S.; Cho, K. Y. Improved electrochromic device performance from silver grid on flexible transparent conducting electrode prepared by electrohydrodynamic jet printing. *J. Mater. Chem. C* **2017**, *5* (48), 12800-12806. DOI: 10.1039/C7TC04840H.

(7) Wu, Z.; Zhao, Q.; Luo, X.; Ma, H.; Zheng, W.; Yu, J.; Zhang, Z.; Zhang, K.; Qu, K.; Yang, R.; et al. Low-Cost Fabrication of High-Performance Fluorinated Polythiophene-Based Vis–NIR Electrochromic Devices toward Deformable Display and Camouflage. *Chem. Mater.* **2022**, *34* (22), 9923-9933. DOI: 10.1021/acs.chemmater.2c01964.

(8) Chaudhary, A.; Pathak, D. K.; Tanwar, M.; Yogi, P.; Sagdeo, P. R.; Kumar, R. Polythiophene–PCBM-Based All-Organic Electrochromic Device: Fast and Flexible. *ACS Appl. Electron. Mater.* **2019**, *1* (1), 58-63. DOI: 10.1021/acsaelm.8b00012.
